# The Use of Prehospital Intensive Care Units in Emergencies—A Scoping Review

**DOI:** 10.3390/healthcare11212892

**Published:** 2023-11-02

**Authors:** Abdullah Alruwaili, Amir Khorram-Manesh, Amila Ratnayake, Yohan Robinson, Krzysztof Goniewicz

**Affiliations:** 1 Emergency Medical Services Department, College of Applied Medical Sciences, King Saud Bin Abdulaziz University for Health Sciences, Al Ahsa 36428, Saudi Arabia; 2 King Abdullah International Medical Research Center, Al Ahsa 36428, Saudi Arabia; 3 Ministry of National Guard—Health Affairs, Al Ahsa 36428, Saudi Arabia; 4 School of Health, University of New England, Armidale, NSW 2350, Australia; 5 Department of Surgery, Institute of Clinical Sciences, Sahlgrenska Academy, University of Gothenburg, 41345 Goteborg, Sweden; amir.khorram-manesh@surgery.gu.se; 6 Center for Disaster Medicine, University of Gothenburg, 40530 Gothenburg, Sweden; yohan.robinson@gu.se; 7 Gothenburg Emergency Medicine Research Group (GEMREG), Sahlgrenska University Hospital, 41305 Goteborg, Sweden; 8 Army Hospital Colombo, Department of Surgery, Colombo 08, Sri Lanka; amila.rat@gmail.com; 9 Swedish Armed Forces Centre for Defence Medicine, 42605 Västra Frölunda, Sweden; 10 Department of Security, Polish Air Force University, 08-521 Deblin, Poland; k.goniewicz@law.mil.pl

**Keywords:** ambulances, intensive care unit, prehospital, treatment, trauma

## Abstract

Background: Amidst a rising tide of trauma-related emergencies, emergency departments worldwide grapple with the challenges of overcrowding and prolonged patient wait times. Addressing these challenges, the integration of prehospital intensive care units has appeared as a promising solution, streamlining trauma care and enhancing patient safety. Nevertheless, the feasibility of such an initiative becomes murky when considered globally. This review delves into the intricacies of prehospital intensive care units’ deployment for trauma care, scrutinizing their configurations, operational practices, and the inherent challenges and research priorities. Methods: A scoping review was performed for eligible studies. The result was uploaded to the RAYYAN research platform, facilitating simultaneous evaluation of the studies by all researchers. Results: A total of 42 studies were initially selected. Four studies were duplicates, and 25 studies were unanimously removed as irrelevant. The remaining studies (n = 13) were included in the review, and the outcomes were categorized into diverse subgroups. Conclusions: A country’s emergency medical services must achieve specific milestones in education, competency, resource availability, and performance to effectively harness the potential of a prehospital intensive care unit. While certain nations are equipped, others lag, highlighting a global disparity in readiness for such advanced care modalities.

## 1. Introduction

Trauma stands as one of the foremost causes of mortality across the globe, a weighty concern for public health and medical communities alike. A substantial number of these mortalities occur in prehospital settings, accounting for more than 50% in the civilian arena and a staggering 90% in military contexts. The ramifications are vast, prompting an urgent reassessment of prehospital emergency care systems [1,2].

As the world rapidly evolves, so does the landscape of prehospital care, presenting an intricate matrix of challenges and innovations for emergency medical services (EMS) worldwide. This constant evolution is punctuated by ground-breaking treatments such as novel bleeding control practices like REBOA (resuscitative endovascular balloon occlusion of the aorta), the introduction of whole blood and blood products, and tranexamic acid [3,4,5,6].

Adding layers to this dynamic milieu are the diverse EMS systems in place across nations. The Franco-German and Anglo-American models exemplify this disparity, each bringing to the fore unique trauma patient management approaches rooted in their intrinsic technological and expertise variances. The Franco-German model, for instance, boasts the advantage of an onboard physician, a factor that potentially tilts the scales in terms of trauma outcomes and other medical conditions [7,8,9].

The introduction of prehospital intensive care units (PICUs) signifies a critical juncture in this narrative. These units, tailored for high-income countries (HICs), stand as an answer to the spiraling demands on EMS—a counterintuitive scenario where there is a surge in non-severe cases even as severe ones are still sparse [9]. The perennial challenge of emergency department (ED) overcrowding, a phenomenon fueled by a confluence of organizational loopholes, ingrained clinical practices, and individual competencies, calls for a fresh examination of prehospital care paradigms [10,11]. The changing role of paramedics and prehospital nurses in this shifting landscape, augmented by the march of mobile technology, signifies a pivotal shift in clinical culture and competencies [12,13,14,15].

Yet, the enticing potential of PICUs comes with its own set of demands—chiefly, a heavy investment in human resources and sustained, rigorous training. United Kingdom-centric research reveals an inconsistent landscape of physician-based prehospital critical care, emphasizing the pivotal role of training in driving positive outcomes [15]. Furthermore, the on-ground realities of the EMS workforce bring to light a contrast: while paramedics bear the brunt of diagnosis and treatment responsibilities, their critical care colleagues possess specialized yet seldom-employed skills—ones that can tip the balance between life and death but also come fraught with risks [9,15,16]. This complexity is epitomized by an expert-driven Delphi study, which painstakingly crafted an emergency care system model, including 177 distinct components, underscoring the intricate nature of emergency care [16].

Crucially, while HICs’ experiences offer invaluable insights, low- and middle-income countries (LMICs) striving to strengthen their EMS and trauma systems must exercise caution. A verbatim adoption of HICs’ guidelines can lead to resource misalignment and budgetary pressures. Striking a balance—one that harmoniously integrates existing resources, involves relevant stakeholders, and champions cost-effective strategies—is critical [5,17,18].

Given the vast complexities and challenges inherent in the realm of prehospital care, this study aims to critically examine the role, functionality, and outcomes of prehospital intensive care units (PICUs) in trauma care. We strive to delve into the structural and operational nuances of PICUs, understand their challenges, find areas of potential research, and evaluate the practicality of their broader adoption across various geographical and economic contexts.

## 2. Materials and Methods

### 2.1. Study Design and Protocol

Following recommendations by Mak and Thomas [19], a scoping review strictly adhering to the PRISMA (Preferred Reporting Items for Systematic Reviews and Meta-Analyses) guidelines was conducted in the search for eligible studies [20]. These guidelines ensured consistency, rigor, and comprehensiveness in our review, resulting in a synthesis of knowledge based on existing or emerging lists of literature on a defined subject.

Creating an expert team with knowledge in trauma and emergency medicine and prehospital care, and after consulting a librarian, the method ensured a map of the extent, range, and nature of the existing literature on the subject and showed the possible gaps after approval of the research question by the expert group.

### 2.2. Search Keywords and the Literature Search Strategy

To ensure a comprehensive and all-inclusive literature search, we opted for a more expansive strategy based on the feedback received. Three main areas to use for the search, i.e., “Prehospital”, “Intensive Care Units”, and “Emergencies”, were chosen by reaching a consensus among experts to answer the study questions, i.e., to critically examine the role, functionality, and outcomes of prehospital intensive care units (PICUs) in trauma care and emergencies.

Using the National Library of Medicine, the MeSH term search was used to apply the most relevant keywords for search in relevant databases [21,22].

1. Prehospital: This word resulted in a diverse combination of emergency care and ambulances, all of which were already included in our search.
2. Intensive Care: This keyword was associated with diverse critical care facilities, such as coronary critical care or pediatric critical care units, none of which were within the scope of this study.
3. Emergency: This keyword resulted in terms related to emergency management of different medical conditions, emergency activities, or special subgroups, e.g., emergency medicine, trauma, etc.

During the search in databases and within each domain, we utilized the “OR” operator to include alternative terms or related keywords. Subsequently, the results of these domains were combined using the “AND” operator.

### 2.3. Databases and Information Sources

After the expert group reached a consensus regarding search strategy and keywords, an initial search was conducted in each database to estimate the number of published articles. PubMed, Scopus, CINAHL, and Web of Science were employed due to their broad coverage of the medical and scientific literature.

### 2.4. Search Strings

#### 2.4.1. The Primary Search String

Initially, the below strategy using OR and AND was applied to each database. For instance, the following shows an example for Medline:

1. “Intensive Care Unit”[Mesh] OR “intensive care”[Title/Abstract] OR “ICU”[Title/Abstract]
2. “Prehospital”[Title/Abstract] OR “Ambulance”[Title/Abstract] OR “Pre-hospital care”[Title/Abstract]
3. “Emergencies”[Title/Abstract] OR “Emergency”[Title/Abstract] OR “Trauma”[Title/Abstract]

Combining the above: #1 AND #2 AND #3

Similar search strings were tailored for other databases like Scopus, CINAHL, and WOS, ensuring the optimization of search results based on each platform’s specific syntax and capabilities.

Appendix A shows the outcomes of using the primary search strings. The results were not manageable since the number of hits per database was proven to be too many to be reviewed. According to guidelines given by several libraries, in such a situation, fewer search keywords should be employed [23]. In this paper, we refined the search and reduced the number of hits by removing similar keywords, which practically meant removing “OR” and keeping the main keywords using only “AND”. In doing so, the final search strings were used in the final search. In this way, we could also harmonize our search keywords and strings since we did not need to alter them for each database. It is well established that each database is different in terms of its interface and functionality. Medline and Scopus, for example, are completely different in terms of searching—Scopus is keyword-only and has no subject headings to search [24].

#### 2.4.2. Final Search Strings

PubMed: “Ambulance” AND “Prehospital” AND “Intensive Care” AND “Unit” AND “Trauma” AND “Treatment”

Scopus: “Ambulance” AND “Prehospital” AND “Intensive Care” AND “Unit” AND “Trauma” AND “Treatment”

CINHAL: “Ambulance” AND “Prehospital” AND “Intensive Care” AND “Unit”

WOS: “Ambulance” AND “Prehospital” AND “Intensive Care” AND “Unit” AND “Trauma” AND “Treatment”

### 2.5. Collaborative Review Tool

After gathering initial search results, data was integrated and imported into RAYYAN, a renowned research collaboration platform fostering remote multinational collaboration [25].

### 2.6. Eligibility Criteria

**Figure 1:**
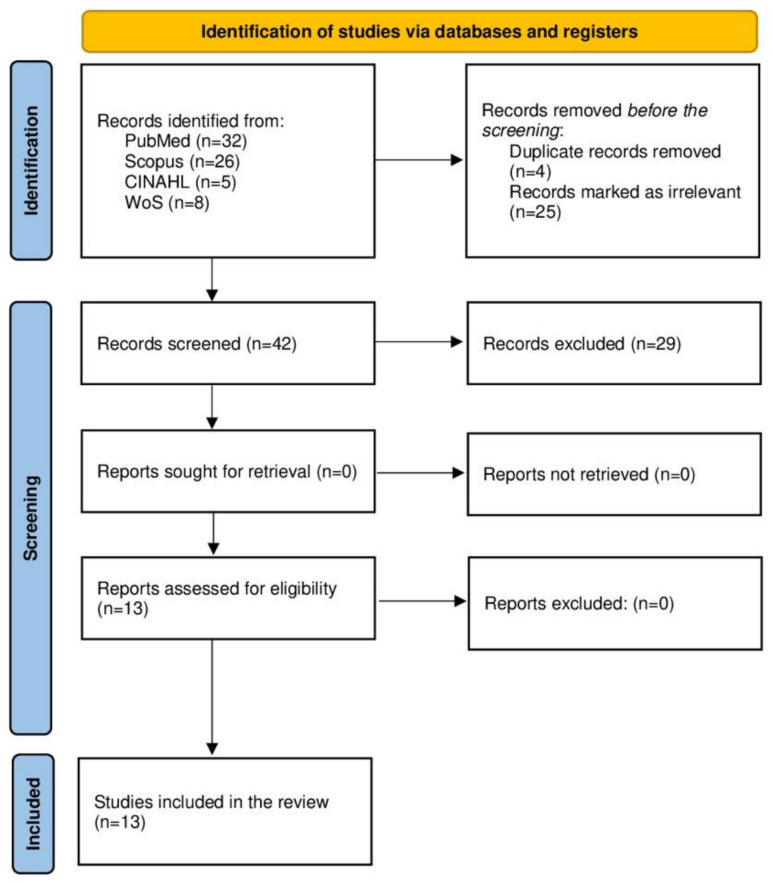
PRISMA flow chart diagram used in this scoping review, which included databases and registers.

We included a diverse range of study designs, focusing on interventions and outcomes in prehospital intensive care, such as advanced airway management, oxygen administration, blood and fluid administration, transportation, and PICU configurations. Discrepancies were resolved by consensus (Figure 1, Supplementary File S1). Included papers must present the related dimensions of PICUs that concern the research question, i.e., configuration, protocol, practical challenges, and challenges. Papers discussing a single diagnosis with no information regarding the main questions were avoided as much as possible.

### 2.7. Inclusion Criteria

The inclusion criteria include articles from 2012 to 2023 in English addressing prehospital intensive care. This timeframe ensured current relevance and manageable volume.

### 2.8. Exclusion Criteria

The exclusion criteria include reports, book chapters, proceedings, and abstracts that were not aligned with our study aims.

### 2.9. Selection of Sources of Evidence

Initially, all abstracts were screened by all authors, and later selected papers’ eligibility and inclusion were thoroughly studied by leading authors. A custom form captured study details and pertinent outcomes. This form was piloted in three studies for efficacy. Data elements included study type, method, authors, origin, publication year, design, duration, setting, PICU specifics, interventions, and results. Primary outcomes, like mortality and functional measures, and secondary outcomes detailing PICU intricacies and research priorities were thoroughly assessed and reported, if available (Supplementary File S2).

### 2.10. Review Process and Data Charting

The result obtained from the search was assessed by two authors. In dubious cases, related articles or other articles citing the article were also reviewed. Once refined and approved by all authors, our collection underwent a review, allowing us to dive deep into each paper’s core insights.

## 3. Results

The primary search resulted in 71 studies: PubMed (n = 32), Scopus (n = 26), CINAHL (n = 5), and Web of Science (n = 8). The first evaluation yielded the removal of 4 duplicates and 25 irrelevant papers. The remaining 42 papers were uploaded to RAYYAN for evaluation by all authors. Another 29 papers were found to be irrelevant. The remaining 13 papers were included in this study (over 75% consensus by all authors). The outcomes were grouped into four subgroups listed below.

The results were reported according to the PRISMA Extension for Scoping Review items [20].

### 3.1. Characteristics of the Reviewed Studies

The reviewed article assessed the patients’ mortality, morbidity, and functional outcomes, control of bleeding, the revised trauma score (RTSc), oxygen saturation, hypoxia, intubation success rate, complications, use of RCC and blood unit waste rates, response time, physician consultation, diagnosis, need for intubation, ethical issues, and decision-making documentation. The findings were organized under four sections: PICU configurations; protocols and practices; benefits of PICU care in patients with trauma; and challenges and research priorities in PICU care for trauma patients. Table 1 shows the characteristics of the included studies, outcomes measured, PICU characteristics, main findings, and comments on the findings (Table 1).

### 3.2. PICU Configurations

#### 3.2.1. Type and Size of the PICU

The findings of this review show that specific aspects of PICU configuration in EMS are varied in different EMS systems and regions. The types, sizes, personnel, training, and equipment in PICU care are varied based on the resources and capabilities of each EMS system. The results showed that PICUs can be configured as air types such as helicopter emergency medical services (HEMS), fixed-wing aircraft, air ambulance vehicles (AAVs), or ground-type EMS (GEMS), including the Mobile Intensive Care Unit (MICU), rapid response vehicles (RRVs), or ground ambulance. For air-type PICUs, HEMS is the most common type that was reported in nine studies from Australia, Germany, Denmark, France, and Japan. Some articles reported HEMS alone [29,30,33,34,38], while four articles mentioned HEMS operating in combination with GEMS [26,27,28,36].

According to Meadley et al. [33], intensive care flight paramedics (ICFPs), who undergo extensive education and training and have clinical skills such as adult and pediatric rapid sequence intubation (RSI), cricothyroidotomy, intraosseous needle insertion, intravenous and arterial cannula insertion, in-field blood gas analysis, blood transfusion, needle thoracostomy, and advanced analgesia, have been used to perform winch operations, which involve extracting patients from inaccessible and austere areas with diverse terrain and climate in Victoria, Australia. MECU is a ground ambulance staffed by a skilled physician and a paramedic who provide advanced life support procedures and resuscitation to critically ill or injured patients or patients with cardiac arrest [32,35,37,39]. Nielsen et al. [37] and Brown et al. [39] reported the use of MECU in Denmark, providing prehospital airway management for unconscious non-trauma patients, while Mikkelsen et al. [35] investigated the outcome of ‘life-saving missions’ by the MECU in Odense, Denmark. The size of a PICU was not explicitly reported in most of the studies (10 out of 13). It refers to the number and capacity of the vehicles or aircraft used for prehospital care. Two other studies [33,34] mentioned that five HEMS were operated across the region of Victoria, Australia. No studies indicated the capacity of PICU vehicles or aircraft used for prehospital care.

#### 3.2.2. Personnel

Findings show that PICUs are staffed by providers who are trained and skilled in advanced life support and other specialized skills. Physicians, specifically anesthesiologists, emergency physicians, and paramedics, are the most reported PICU staff. Studies also reported paramedics with specialized training, including intensive care paramedics (ICPs) and intensive care flight paramedics (ICFPs), emergency medical technicians (EMTs), emergency nurses, and aircrew as PICU staff. In most of the studies, physicians attended patients alone or together with other crew members or physicians [26,36,37]. In addition, physicians also supervise [29] or consult paramedics and approve treatment provided by them [30,34]. Some studies reported that the presence of expert physicians in a prehospital setting was found to be beneficial for the survival of patients with cardiac arrest, who need respiratory support, and those with trauma (85% of 701 included patients) [35,37].

#### 3.2.3. Training

Personnel working in PICUs in EMS undergo specialized training in prehospital critical care. They are trained to provide advanced life support interventions, manage critical conditions, and stabilize patients during transport. Moreover, providers were trained to provide basic or intermediate care to trauma patients, such as oxygen administration, vascular access, and defibrillation [33,39]. The most reported advanced skill training includes advanced airway management, cricothyroidotomy, and advanced life support training [27,29,30,37,39]. In addition, some ICFPs had additional qualifications or skills to perform advanced care such as intubation or blood transfusion [30,33,34,39]. Hansen et al. [28] reported that physicians working on MECU had sub-specialized training in prehospital critical care. Findings also show that most life-saving interventions provided in prehospital settings exceed the competencies of the EMT or PM [35]. On the other hand, studies also reported that interventions that require advanced skills are less performed in the scene [33]. All providers in PICUs need to be authorized and pass regular skill reaccreditation [33,34].

#### 3.2.4. Equipment

Studies have reported various types of medical equipment that are typically found in PICUs. They are equipped with advanced medical equipment and monitoring devices to provide comprehensive care during transport. This includes airways, vascular access devices, cardiac monitors, defibrillators, infusion pumps, eFAST/FAST, ultrasonography, and other life-saving equipment necessary for critical care interventions. However, some of the equipment is specific to certain types of PICU or interventions, such as extraction devices or pigtail catheters [33,38,39]. The most common were airway devices that mainly advanced airways [29,33,36], followed by blood product sets [25,33].

### 3.3. Protocols and Practices of the PICU

#### 3.3.1. Protocols

Studies reported various protocols and guidelines to guide the dispatch, triage, assessment, and treatment of patients in the prehospital setting [27,28,29,30,32,34,35,39]. These included clinical practice guidelines, standard trauma care protocols, criteria-based protocols, standard operating procedures, competence-based practice, or injury classification protocols [28,30,34,35]. Some protocols were common to most types of PICU, while others were specific to certain types of PICU or interventions [27].

Documentation of patient information was often performed using regional or national registries or databases, such as electronic patient care records or databases, quality assurance databases, or MECU databases [27,28,29,30,32,33,34,35,39]. However, there were inconsistencies in documentation across the studies, and some studies reported missing data linkage or inconsistent charting [36,37].

#### 3.3.2. Dispatch Process and Practice

The PICU dispatch process varied among the studies, depending on the context, the type, and the severity of the patient’s condition [27,32,35]. There were different dispatch systems used, such as a nationwide Emergency Medical Dispatch (EMD), a criteria-based dispatch system, and a computer-aided dispatch system [27,28,32,39]. The PICU service was activated based on predefined criteria or information from the caller or by request from the treating medical team or the emergency medical technicians (EMTs) in the primary ambulance [27,28,32,35,39]. However, studies did not report the dispatch process or criteria at all, while others reported some information on the mission duration or dispatch operation procedures [26,29,30,33,34,36,37,38].

#### 3.3.3. Assessment and Triage

The triage and assessment of patients in the PICU were mainly based on physiological parameters such as respiratory rate, systolic blood pressure, heart rate, Glasgow Coma Scale (GCS), shock index, or injury severity score (ISS). Studies also reported the use of anatomical triage, ultrasound assessment, focused assessment with sonography for trauma (FAST), or return of spontaneous circulation (ROSC) to evaluate the patient’s condition [28,29,30,32,33,34,35,38,39]. The assessment was often graded into categories or scales to indicate the severity of the patients [28,32,33,35]. The use of anatomical triage and the Australian triage scale were the only triage approaches reported by two studies [28,39].

#### 3.3.4. Clinical Procedures

Findings show that endotracheal intubation, to secure the airway of trauma patients, was the most reported clinical procedure performed by the PICU [26,30,32,36,37]. Other clinical procedures reported included blood transfusion, rapid sequence intubation (RSI), extraction devices, and other diagnostic or therapeutic procedures using ultrasound or pigtail catheters [27,30,36]. Some procedures are specific to certain contexts or cases, such as pericardial drainage in a “doctor helicopter” for multiple trauma cases with pericardial tamponade [38] and winch extraction to extricate and transfer patients from remote or inaccessible locations [33]. One study reported that PICUs were involved in mass casualty incident (MI) management, though they only scooped and ran, and no other clinical interventions or transfers were provided in prehospital settings [28].

#### 3.3.5. Communication and Transportation

Radio communication was used to coordinate with dispatch centers, receiving hospitals, or emergency services in some studies [27,28,32,38]. According to Hansen et al. [28], EMS uses a unified radio communication channel during MI or an otherwise independent communication channel. Regarding transportation, a primary response, i.e., transferring patients from the scene to the hospital, is the most commonly reported, except in two studies that reported interfacility transfers [33,38]. The transportation type, time, and distance were dependent on the location and availability of the service, and most of the patients were transported by GEMS rather than HEMS [26,36].

### 3.4. Benefits of PICUs in Trauma Care

Prehospital ICUs (PICUs) can have benefits for trauma patients compared to standard ambulances. PICUs can improve survival, reduce complications, and enhance the quality of care for some trauma patients [32,33,38]. One study reported that the frequency and duration of hypoxia in the post-intubation period were reduced after the application of apneic oxygenation in patients with severe trauma [30]. There was a statistically significant benefit from apneic oxygenation in reducing the frequency of peri-intubation hypoxia (SpO2 ≤ 90%) for patients with initial SpO2 > 95%. Another study found that paramedics administering RCCs in a prehospital setting is feasible and can improve median systolic blood pressure and shock index. There were no transfusion-related complications identified, while further research suggested that optimal use of resuscitative fluids is warranted [29]. Another study reported that prehospital medical management in trauma patients is associated with a reduction in 30-day mortality, and direct transfer of the casualties by HEMS (SMUR helicopter) to a trauma center is also associated with a decrease in mortality risk [36]. A case report of the successful treatment of blunt traumatic cardiac tamponade in a 55-year-old man also reported that the out-of-hospital pericardial drainage in a “doctor-helicopter” ambulance saved the patient’s life [38].

### 3.5. Challenges and Research Priorities for PICUs in Trauma Care

Prehospital intensive care units (PICUs) face several challenges in trauma care, which potentially need to be prioritized in research. Some of the challenges reported by the studies include the implementation, evaluation, and improvement of PICU practices [28,37,38]. For instance, a data linkage study in Australia found that prehospital Code Crimson (CC) activation was highly specific to the need for hemorrhage control intervention in hospitals [27]. The study suggested the need to improve the sensitivity of prehospital CC activation and further research on criteria to triage and select patients most likely to benefit from intervention. Moreover, there are also communication gaps and safety threats to EMS personnel during MI response, while the role of PICUs during such incidents needs further research [28]. There were also gaps in ethical decision-making documentation in prehospital life-and-death situations, which needs the implementation of a standard template in the prehospital medical records [32]. Another study also found that most of the treatment necessary to save the patient’s life was administered out of the competence of the attending EMT or PM and argued that specialists in anesthesiology should be applied in the prehospital setting to provide advanced procedures [35]. Therefore, there is a need to provide further education and training to EMTs and PMs to improve their competencies and skills in prehospital care. The study also recommended that further education and training should be provided to EMTs and PMs to improve their competencies and skills in prehospital care. The study also reported that the majority of trauma patients had low-acuity injuries, and thus there is a need to focus research, training, and resources solely on high-acuity patients, which will not cater to the needs of the majority [39]. As most of the studies were limited in design, there is a need for further research with a strong design or large sample size to identify the potential benefits associated with interventions in different subgroups [26,27,29] or long-term complications with the treatments [30,34].

## 4. Discussion

This study set out to probe the intricacies of PICU use for trauma patients, encompassing configurations, practices, and the myriad challenges that arise, especially within the context of diverse nations. Consistent configurations and practices for PICUs remain elusive, largely due to the variable prerequisites across nations and the four crucial pillars of surge capacity: staff, supply, space, and system [40].

Historical military experiences provide deep insights into prehospital care, emphasizing the necessity for swift, efficient interventions in chaotic and challenging situations in both civilian and military settings. These challenges create opportunities for civilian–military collaboration and exchange of knowledge, such as the use of tourniquets, point-of-care ultrasound (POCUS), and REBOA [31,41,42,43]. These military–civilian medical translations underscore the vitality of taking lessons from battlefields and contextualizing them for broader emergency care scenarios. It also lends credence to the PICU concept, which, though debated, thrives in military contexts due to rigorous system thinking and guidelines [43,44]. In the civilian sector, the PICU’s merits are evident, but they come at significant financial and training costs, making their global feasibility, especially in LMICs, uncertain [5,31,41,42]. While innovations like telemedicine and artificial intelligence offer potential transformations for PICUs, vital procedures such as REBOA come at steep costs, prompting discussions about their cost-effectiveness amidst technological growth and research gaps [45,46].

Considering the geographical landscape, one must evaluate if the nation’s topography complements or hampers PICU implementation. For instance, Norway’s unique geography leans more towards HEMS investment, while Sweden can use both GEMS and HEMS, making decisions rooted in cost-effectiveness. Policy decisions like “scoop and run” or “stay and play” also influence these choices, with underlying financial implications [47,48]. The requirement of staff skills and knowledge, in turn, hinges on geography, population density, and infrastructure. Complex medical conditions coupled with prolonged transportation mandate a more advanced care system and another financial commitment. The inclusion of a physician onboard can significantly affect the cost structure, despite the debatable necessity given certain contexts [8]. Therefore, the type of EMS (GEMS, HEMS, or MEMS) a country chooses is a significant financial commitment, and its utility must be weighed against its cost.

The often-overlooked mental health dimension of trauma care also merits attention. Combat settings reveal the profound psychological effects of trauma. Similarly, in civilian care, it is vital to address both the physical and psychological impacts of trauma. PICU settings must integrate psychological care for comprehensive recovery. Additionally, considering the mental well-being of the constantly stressed PICU staff is crucial for consistent quality care. This comprehensive approach demands both financial and integrated strategies to cater to patients’ and staff’s holistic needs [49].

The rising violence brings trauma care into sharp focus. While complex injuries demand specialized care and financial outlays for training and equipment, post-intervention quality of life is paramount. Beyond immediate PICU responses, trauma care’s long-term financial implications are significant. Emphasizing rehabilitation, as seen in military contexts, becomes crucial. Such practices in civilian care point towards tailored rehabilitation programs for trauma patients, balancing their potential benefits against the PICU’s heightened costs [50]. Although studies, such as the one comparing two European countries, namely Germany and Switzerland, have shown that despite differences in the type of staffing and organizations, the standard mortality rates in prehospital settings can be similar [51], and despite the rare need for specialized PICU interventions [28,33,38], their financial justification is vital, and any investments in PICUs (staff, supplies, space, and system) must encompass educational initiatives and accessible training programs.

However, standardizing these across diverse medical landscapes is challenging. While comprehensive training raises costs, it ensures improved patient safety and care quality, potentially leading to better patient outcomes [52,53]. For instance, military medical practices emphasize the importance of strict triage protocols to strengthen the compatibility between prehospital and hospital arenas and maximize resource efficiency and survival rates. Incorporating such triage in civilian PICUs may enhance their cost-effectiveness [54]. The significant financial commitment to PICUs, with start-up costs in HICs ranging between $1.5 and $3 million [55] and operational costs like the UK’s HEMS exceeding £2 million annually [56], raises ethical concerns, especially when compared to more affordable care settings. Such financial considerations are critical, especially in resource-limited countries.

Policymakers worldwide face the challenge of integrating these findings into their health strategies. The question arises: Should there be a push for more standardized PICU guidelines on a global scale? While HICs might be poised to adopt and adapt, supporting LMICs is vital. Potential solutions could involve international collaborations, funding, and knowledge-sharing to bridge the gap. LMICs, facing economic constraints, can investigate models of shared resources or regional PICUs to ensure accessibility without bearing the burden of individual setups.

## 5. Limitations

The review was constrained to studies published in English, which may have inadvertently excluded pertinent research and insights available in other languages, creating potential gaps in capturing a comprehensive global perspective on PICUs, mainly an underrepresentation of studies from certain geographical regions and LMICs.

The inclusion criteria used for this study were deliberately stringent and designed to streamline the volume of studies for a more focused review. However, this approach could mean broader or varied terminologies not included in the primary search criteria might have been overlooked, excluding certain relevant studies.

Additionally, while this study did endeavor to incorporate relevant studies from the reference lists of included papers, this method inherently relies on the comprehensiveness of the original papers’ bibliographies; any omission in their lists could indirectly lead to an omission in our review.

This review is also temporally bound, capturing insights available up to its execution, and might not include the most recent advancements or findings in the realm of PICUs, especially given the rapid pace of medical and technological advancements. Moreover, while the review extensively delved into the financial and ethical dimensions of PICUs, other vital aspects such as long-term patient outcomes, clinical effectiveness, or system-wide impacts might not have received similar in-depth exploration.

While we acknowledge the breadth and depth of insights provided in our review, a predominant number of studies hail from specific geographical regions. This inevitably means that the experiences, challenges, and innovations occurring in certain parts of the world, particularly in regions that may not publish extensively in English, are not adequately represented. This geographical bias can skew the perception of PICU usage and best practices, limiting the universality of our findings. Future reviews might benefit from a more inclusive approach that delves into the literature in multiple languages and specifically targets underrepresented regions to provide a more holistic view of PICUs in emergencies globally.

## 6. Conclusions

The evolving realm of prehospital intensive care units (PICUs) faces considerable disparities in standardization, with a glaring absence of universally endorsed guidelines governing their design, staffing, usage, and proliferation. While there is no disputing the pivotal role they play in specialized trauma cases, the towering costs associated with their establishment and maintenance invite profound financial and ethical quandaries. These challenges are magnified when juxtaposing the infrastructural and financial capacities of high-income nations against those of low- and middle-income countries. This examination accentuates an urgent call for rigorous research dedicated to PICUs. Such research should not only delineate the efficacy and outcomes associated with these units but also delve deep into their financial implications and the ethical challenges they pose. Ultimately, the aspiration should be to harmonize the imperatives of exemplary patient care with the principles of economic viability and ethical responsibility.

## Figures and Tables

**Table 1:** Characteristics of included studies, measured outcomes, PICU characteristics, main findings, and comments.

Author (YOP)	Study Objectives	PICU Configuration *	Criteria for Activation	Beneficiary/Target Patient Population	Performed Procedure Enroute (on Field)	Outcome	Risk–Safety	Comments
Andruszkow (2016) [26]	- Examine whether age, gender, injury mode/severity would help identify trauma patient populations who might benefit explicitly from HEMS rescue.	- HEMS and GEMS physicians are trained standardly in ATLS and PHTLS in Germany.	NR	Special subgroups: - middle-aged and older patients (>55 years) - low-energy trauma - minor-severity injuries These subgroups witnessed the best survival potential provided by HEMS.	Six on-scene procedures were documented in the TR-DGU: - intubation - chest tube insertion - vasopressors, sedatives, or volume infusion - CPR	- HEMS rescues improve overall survival compared to GEMS.	NR	NR
Partyka (2016) [27]	- Describe the clinical characteristics of patients who had PH trauma CC activation in the first 23 months of a statewide policy implementation (main outcome: hemorrhage control). - Compare these characteristics to trauma patients transported by retrieval services who met the CC criteria but whose PH CC was not activated (“missed code crimson”).	NR	NR	- Code Crimson-activated patients had more multisystem trauma (80%), especially thoracic trauma (hemopneumothorax, multiple rib fractures, pulmonary contusion) and femoral fractures, compared with the missed CC patients. - Greater degree of hemodynamic instability in the CC-activated group, with a higher shock index.	- intubation (60/72) - chest decompression (39/72) - positive eFAST (30/72) - blood products consistent with CC criteria (71/72)	- In-hospital mortality rate was lower in CC-activated patients (20% vs. 33–48%).	NR	NR
Hansen (2016) [28]	- Describe immediate PH EMS response to the Great Belt Train Accident. - Evaluate adherence to guidelines to identify areas for improvement for future MI management.	- The EMS in Denmark is a three-tiered system consisting of ambulances manned by a combination of EMT with basic, intermediate, or PM levels of training. - The Danish EMS includes rapid response units manned by PMs and MECUs staffed by specialists in anesthesiology with a sub-specialization in PH critical care. - A supplementary physician-manned HEMS is also available.	- EMS response relies on a systematic criteria-based dispatch protocol.	- No patients required transport over longer distances, and their injuries did not require extensive medical treatment at the scene. Therefore, HEMS helicopters were cancelled on-scene.	- 2 rescue teams (1 EMS physician and 2 EMT/PMs) entered the train from the east end and triaged the patients inside the coaches as per physician discretion using anatomical triage. - Transition from the pre- to in-hospital phase in MI should be seamless, which requires predefined plans and systems for trauma management.	- EMCC physician decided to allocate all passengers to the same hospital once the Medical Incident Commander established the magnitude of the MI. - Reports from other major European incidents underline command and control in every phase as an essential component.	- Weather conditions may potentially compromise EMS personnel’s safety and influence patient transfers from the accident site.	- Time from dispatch to arrival was compromised because of traffic congestion on both sides of the connection.
Crewdson (2016) [29]	- Investigate whether the introduction of apneic oxygenation would reduce the frequency of desaturation in trauma patients undergoing prehospital emergency airway management.	- Doctor–PM teams are delivered by helicopter and fast-response cars. - Flight PMs in the ambulance control room dispatch services (target critically ill or injured patients). - A ground ambulance is also always dispatched.	- The ambulance control room dispatches the services, and specific criteria target critically ill or injured patients.	- Advanced airway interventions are necessary for a small subgroup of severely injured patients.	- Nasal oxygenation using low-flow nasal prongs is a low-risk, easily administered procedure for passive apneic oxygenation in the pre-intubation and peri-intubation phases of emergency anesthesia.	- Apneic oxygenation reduced the frequency of peri-intubation hypoxia (SpO2 ≤ 90%) in patients with initial SpO2 > 95% (*p* = 0.0001). - Also, the recovery phase improved in patients with severe hypoxia prior to intubation.	- Peri-intubation hypoxia (SpO2 ≤ 90%) is documented in prehospital advanced airway interventions (the most frequent AE). - Hypoxia occurred in 9.2% of patients during the first attempt at intubation in an emergency setting, reaching 37.8% upon repeated intubation.	NR
Heschl (2016) [30]	- Describe mortality and functional outcomes after 6 months in children with TBI (PH RSI by HEMS PMs vs. no intubation).	- Ambulance Victoria provides road ambulances and 5 emergency helicopters throughout the state. - The system is two-tiered, with ALS PMs and/or ICPs. - ICPs are trained to perform ETI in adults and pediatrics (i.e., respiratory arrest, cardiac arrest, and impaired consciousness).	NR	- Pediatric patients with suspected TBI.	- PM airway management includes ETI without drugs or RSI.	- No difference in the unadjusted mortality rate or functional outcome after 6 months between both groups.	- Intubation success rate was 99% (86/87), with a first-pass success rate of 93% (81/87).	NR
Brown (2016) [31]	- Describe the epidemiology of trauma attended by PMs in Western Australia. - Describe trauma incidence and mortality rates and trends. - To compare the characteristics of patients (died at the scene vs. died on the day of injury vs. died within 30 days of the event vs. survived 30 days). - To report interventions performed by PMs.	- St. John Ambulance WA within the metropolitan area: ambulances are staffed by PMs providing PH care (i.e., ALS), guided by CPG.	NR	- Patients transported with the highest acuity level accounted for 2.7%.	- ALS (i.e., endotracheal intubation, surgical).	NR	NR	- The lack of PM exposure to high-acuity patients, the resulting skill decay, and decreasing job satisfaction have previously been reported.
Mikkelsen (2016) [32]	- Investigate to what extent ethical considerations are documented in discharge summaries in cases of life-and-death decisions made by emergency care anesthesiologists in a Danish PH setting. - Describe the nature of such considerations and seek the establishment of suggested recommendations.	- MECU in Odense is a part of a three-tiered system that supplements an ordinary ambulance manned by two EMTs or an ambulance assisted by a PM. - It consists of 1 rapid-response car, operating all year round, manned by a specialist in anesthesiology and an EMT.	NR	- Patients in cardiac arrest.	- In Denmark, as in most other countries, physicians are responsible for the act of declaring a patient dead in the PH field.	- In most cases where ethical content was identified, ethical considerations led to a decision to terminate treatment.	NR	- PH physicians may face ethical dilemmas in life-and-death decisions. - An EMT or PM is obliged to initiate resuscitative efforts for all lifeless patients until declared dead by a physician. - All lifeless patients are legally regarded as not dead but as patients with cardiac arrest.
Meadley (2016) [33]	- Define the characteristics of winch missions undertaken by ICFPs in Victoria, Australia, with a focus on extraction methods and clinical care delivered at the scene.	- All 5 aircraft (4 Bell 412EP and 1 air-bus Dauphine N3) are capable of winch operations, operating 24/7.	NR	- 109 (87.2%) patients experienced trauma with a mean revised trauma score of 7.52. - Isolated limb fractures were the most common injuries, whereas falls and vehicle-related trauma were the most common mechanisms of injury.	- Vascular access (38.4%), analgesia (44.0%), and anti-emetic administration (28.8%) were the most common interventions.	NR	- Winch benefits must be weighed against the risk of injury and/or fatality to both crew and patients. - Training for and maintaining updated winch operations incurs a significant financial and operational burden.	NR
Heschl (2016) [34]	- Describe the implementation and initial experience of RCC administered in a PM-staffed HEMS in Victoria, Australia.	- Primary missions: helicopters staffed with one pilot, an air crewman, and an ICFP. - The latter undergo extensive training to be qualified to treat critically ill patients compared with road-based PMs. - Their skillset includes advanced airway management by RSI and cricothyroidotomy, comprehensive analgesia (i.e., opioids, ketamine), and the administration of vasoactive medications. - HEMS carries point-of-care devices to measure ABG and Hb.	NR	- 150 patients received PH RCCs, of which 136 suffered trauma. - 66.7% of them were males, and 62.5% of them were involved in car accidents. - 97.4% had an ISI ≥ 12.	- Advanced airway management (RSI), cricothyroidotomy, analgesia (i.e., opioids, ketamine), and vasoactive drug administration.	- SBP (80 mmHg vs. 94 mmHg, *p* < 0.001) and shock index (1.50 vs. 1.23, *p* < 0.001) improved between the time of consultation and arrival at the hospital. - Trauma-related mortality was 37.7%. - No transfusion-related complications were identified.	- To assure optimal safety, consultation was required with an ARV physician coordinator prior to the administration of RCC. - Main indication for RCC administration was refractory hypovolemic shock after 40 mL/kg administration of crystalloid fluids. - Each helicopter carries 4 RCC units equipped with a temperature data logger that records the temperature every minute. - Maintenance of storage temperature 2–8 °C is achieved by refrigerated gel pads (exchanged twice daily).	NR
Mikkelsen (2016) [35]	- To determine patients’ survival between a specialized physician at the scene and an EMT or PM.	- Anesthesiologist-administered PH therapy increases the level of treatment modalities. - In May 2006, a MECU was initiated in Odense, Denmark, consisting of a rapid-response car operating all year round. It is manned by a specialist anesthesiologist and an EMT. - It operates as part of a two-tiered system in which the MECU supplements an ordinary ambulance manned by 2 EMTs.	-The criteria for denoting a case as ‘patient undergoing life-saving measures’ included: Explicit criteria: - Intubation or other airway procedures exceeding the competence of the PM or EMTs. - Advanced medical treatment exceeding the competence of the PM or EMTs in cardiac arrest and/or defibrillation. Implicit criteria: - Advanced medical treatment and fluid resuscitation exceeding the competence of the attending PM in severe shock states and severe hypovolemia, respectively.	- Survivors of critical illness or those with post-intensive care syndrome (most benefited from peer support groups).	- Intubation/other airway procedures exceeding the competency of PMs or EMTs. - Advanced medical treatment exceeding the competence of the PM or EMTs in cardiac arrest and/or defibrillation when indicated by the attending physician. Implicit criteria: - Advanced medical treatment and fluid resuscitation exceeding the competence of the attending PM in severe shock states and severe hypovolemia, respectively.	- 37.8% of patients were discharged to their own homes following in-hospital treatment.	NR	NR
Tissier (2016) [36]	- Study the impact of medical prehospital management (SMUR vs. fire brigades) on the 30-day mortality of severe blunt trauma victims. - Examine the influence of the mode of transport by SMUR (air vs. ground ambulance). - Evaluate patients’ outcomes based on (1) the level of hospitalization at the time of first admission and (2) the pattern of early surgical and medical procedures. - Evaluate the use of the motor component of GCS on the performance of prehospital triage scores.	NR	NR	- Heli-SMUR patients were more severely injured, but the ISS was similar between groups. - Hypotension and severe spinal injuries were more frequent in heli-SMUR patients than in ground-SMUR patients.	- SMUR allows a variety of therapeutic strategies involving ALS care. - EPs can perform rapid sequence induction before endotracheal intubation for mechanical ventilation, sedative and analgesic drug administration, pleural exsufflation, appropriate fluid loading, and osmotherapy.	- Risk of death before ICU discharge (within 30 days) was significantly lower for heli-SMUR patients than for ground-SMUR patients after adjustment of initial status, ISS, and overall surgical procedures.	- The median time to hospital admission was higher for heli-SMUR than ground-SMUR patients (2.3 vs. 1.8 h, respectively).	NR
Nielsen (2016) [37]	- Describe the experience of airway management in unconscious non-trauma patients in the PH setting with a physician-manned MECU. - Main outcome: the need for subsequent tracheal intubation during hospitalization after initial treatment.	- MECU is manned by a team consisting of a medical doctor and a specially trained ALS provider. - Doctors on MECUs are all specialists in anesthesiology, with at least 7 years of postgraduate experience and a minimum of 5 years of specialty training.	NR	- Unconscious (GCS scores < 9) non-trauma patients.	NR	NR	NR	NR
Otsuka (2016) [38]	- A report of a blunt-trauma patient’s life by diagnosing traumatic cardiac tamponade and performing immediate pericardial drainage in a doctor-helicopter.	- PH diagnosis of cardiac tamponade and performance of pericardial drainage by a skilled EMP transported to the field by doctor-helicopter ambulance, followed by transportation of patients to a critical care center, may prevent PH deaths from cardiac trauma.	NR	- Blunt-trauma patient’s life by diagnosing traumatic cardiac tamponade.	- Immediate pericardial drainage using a portable US device at the heliport prior to transfer.	- Save this blunt-trauma patient’s life by diagnosing traumatic cardiac tamponade and performing immediate pericardial drainage using a portable US at the heliport prior to hospital transfer.	NR	- Cardiac tamponade is one of the main causes of death before hospital arrival in cases of chest trauma; late diagnosis and treatment can be fatal. - FAST is useful for the diagnosis of cardiac tamponade, and a portable US device can be used anywhere.

* PICU configuration includes: platform (air vs. group), physicians/paramedics, equipment/possible interventions, and EMS systems. HEMS: Helicopter Emergency Medical Service; PH: Prehospital; CC: Code Crimson; EMS: Emergency Medical Services; MI: Myocardial Infraction; TBI: Traumatic Brain Injury; ICFP: Intensive Care Flight Paramedics; RCC: Red Cell Concentrate; PM: Paramedic; GCS: Glasgow Coma Scale; MECU: Mobile Emergency Care Unit; ATLS: Advanced Trauma Life Support; PHTLS: Prehospital Trauma Life Support; EMTs: Emergency Medical Technicians; ICPs: Intensive Care Paramedics; ALS: Advanced Life Support; CPG: Clinical Practice Guidelines; RSI: Rapid Sequence Intubation; ABG: Arterial Blood Gases; Hb: Hemoglobin; EMP: Emergency Medicine Physician; ISS: Injury Severity Score; US: Ultrasound; ETI: Endotracheal Intubation; CRP: Cardiopulmonary Resuscitation; SBP: Systolic Blood Pressure; NR: Not Reported; GEMS: Ground Emergency Medical Services; SMUR: Medical-Staffed Emergency Mobile Unit; YOP: Year of Publication; eFAST: Focused Assessment with Sonography for Trauma; SAR: Search and Rescue.

## Data Availability

The datasets used and/or analyzed during the current study are available from the corresponding author upon reasonable request. The necessary information is available in Appendix A.

## Supplementary Materials

The following supporting information can be downloaded at: https://www.mdpi.com/article/10.3390/healthcare11212892/s1, File S1: Final Search Strategies, Selection, and Coding; File S2: Assessment and report of primary and secondary outcomes.

## Appendix A. The Primary Search Strategy and Its Outcomes

**Search strategy according to the reviewer’s request**

“Intensive Care” OR “intensive care” OR “ICU” AND “Prehospital” OR “Ambulance” OR “Prehospital Care” AND “Emergencies” OR “Emergency” OR “Trauma”

**PubMed: 19,503; AND 1248; AND 890,364 hits**

Stepwise add the search terms and reduce the number of hits. 1248 hits if we do not add the last search Keywords.

https://pubmed.ncbi.nlm.nih.gov/?term=%22Intensive+Care%22+OR+%22intensive+care%22+OR+%22ICU%22+AND+%22Prehospital%22+OR+%22Ambulance%22+OR+%22Prehospital+Care%22+AND+%22Emergencies%22+OR+%22Emergency%22+OR+%22Trauma%22&sort=date&size=50 (accessed on 10 August 2023, for all).

**Scopus: 3178 hits**

Remember that Scopus is only based on keywords. Stepwise add the search terms and reduce the number of hits.

https://www-scopus-com.ezproxy.ub.gu.se/results/results.uri?sort=plf-f&src=s&st1=%22Intensive+Care%22+OR+%22intensive+care%22+OR+%22ICU%22+AND+%22Prehospital%22+OR+%22Ambulance%22+OR+%22Prehospital+Care%22+AND+%22Emergencies%22+OR+%22Emergency%22+OR+%22Trauma%22&sid=b4044ff6aa10d739ba866e0ea9821d2c&sot=b&sdt=b&sl=160&s=TITLE-ABS-KEY%28%22Intensive+Care%22+OR+%22intensive+care%22+OR+%22ICU%22+AND+%22Prehospital%22+OR+%22Ambulance%22+OR+%22Prehospital+Care%22+AND+%22Emergencies%22+OR+%22Emergency%22+OR+%22Trauma%22%29&origin=searchbasic&editSaveSearch=&yearFrom=Before+1960&yearTo=Present&sessionSearchId=b4044ff6aa10d739ba866e0ea9821d2c&limit=10

**CIAHL: As title: 4349, and as abstract: 10,943**

Can use title, abstract, or neither in the search. Fewer and more reliable hits if nothing was chosen, even if it initially had higher hits.

“Intensive Care” as title: 4342 hits, and as abstract: 10,857, and none: 19,697

https://web-s-ebscohost-com.ezproxy.ub.gu.se/ehost/resultsadvanced?vid=3&sid=6a040807-bcba-4465-9dd2-bb36e4f0166e%40redis&bquery=TI+%22Intensive+Care%22&bdata=JmRiPWM4aCZ0eXBlPTEmc2VhcmNoTW9kZT1BbmQmc2l0ZT1laG9zdC1saXZl

or

https://web-s-ebscohost-com.ezproxy.ub.gu.se/ehost/resultsadvanced?vid=4&sid=6a040807-bcba-4465-9dd2-bb36e4f0166e%40redis&bquery=AB+%22Intensive+Care%22&bdata=JmRiPWM4aCZ0eXBlPTEmc2VhcmNoTW9kZT1BbmQmc2l0ZT1laG9zdC1saXZl

AND

“Prehospital Care” as title: 2, and as abstract: 56, and none: 47

https://web-s-ebscohost-com.ezproxy.ub.gu.se/ehost/resultsadvanced?vid=9&sid=6a040807-bcba-4465-9dd2-bb36e4f0166e%40redis&bquery=%22Intensive+Care%22+AND+%22prehospital+care%22&bdata=JmRiPWM4aCZ0eXBlPTEmc2VhcmNoTW9kZT1BbmQmc2l0ZT1laG9zdC1saXZl

AND

“Emergency” as title: 5, and as abstract: 30, and none: 18

https://web-s-ebscohost-com.ezproxy.ub.gu.se/ehost/resultsadvanced?vid=10&sid=6a040807-bcba-4465-9dd2-bb36e4f0166e%40redis&bquery=%22Intensive+Care%22+AND+%22prehospital+care%22+AND+%22Emergency%22&bdata=JmRiPWM4aCZ0eXBlPTEmc2VhcmNoTW9kZT1BbmQmc2l0ZT1laG9zdC1saXZl

AND

“Trauma” None: 10, none of them are really about PICUs

https://web-s-ebscohost-com.ezproxy.ub.gu.se/ehost/resultsadvanced?vid=11&sid=6a040807-bcba-4465-9dd2-bb36e4f0166e%40redis&bquery=%22Intensive+Care%22+AND+%22prehospital+care%22+AND+%22Emergency%22+AND+%22TRauma%22&bdata=JmRiPWM4aCZ0eXBlPTEmc2VhcmNoTW9kZT1BbmQmc2l0ZT1laG9zdC1saXZl

**Web of Science**

“Intensive Care” OR “intensive care” OR “ICU” Hits: 275,045

https://www-webofscience-com.ezproxy.ub.gu.se/wos/woscc/summary/d98d0fbc-f9ad-434b-9068-caf5fc9533bb-ab19fdab/relevance/1

AND “Prehospital” OR “Ambulance” OR “Prehospital Care” Hits: 255,657

https://www-webofscience-com.ezproxy.ub.gu.se/wos/woscc/summary/c35054c4-c1b3-4183-a5ed-6a340f142ca8-ab1a21ee/relevance/1

AND “Emergencies” OR “Emergency” OR “Trauma” Hits: 1,150,240

https://www-webofscience-com.ezproxy.ub.gu.se/wos/woscc/summary/c5b37b4b-d443-4f77-aaaf-79593baa6830-ab1a3168/relevance/1
