# Supplementary material for: The Use of Prehospital Intensive Care Units in Emergencies—A Scoping Review
Source: Healthcare (Basel). 2023 Nov 2;11(21):2892. doi: 10.3390/healthcare11212892 (PMC10647734; doi:10.3390/healthcare11212892)
Supplement: Supplementary file 1 [file healthcare-11-02892-s001.zip › Supplementary File S1.pdf]

**Supplementary File S1. Final Search Strategies, Selection, and Coding**

**<u>Initial Screening PICU</u>**

**Search string**

**PubMed n = 32:** “Ambulance” AND “Prehospital” AND “Intensive Care” AND “Unit” AND “Trauma” AND “Treatment”

https://pubmed.ncbi.nlm.nih.gov/?term=%22Ambulance%22+AND+%22Prehospital%22+AND+%22Intensive+Care%22+AND+%22Unit%22+AND+%22Trauma%22+AND+%22Treatment.%22&sort=date&size=50

**Scopus n = 26:** “Ambulance” AND “Prehospital” AND “Intensive Care” AND “Unit” AND “Trauma” AND “Treatment”

https://www-scopus-com.ezproxy.ub.gu.se/results/results.uri?sort=plf-f&src=s&st1=%22Ambulance%22+AND+%22Prehospital%22+AND+%22Intensive+Care%22+AND+%22Unit%22+AND+%22Trauma%22+AND+%22Treatment.%22&sid=9b329ac8a4f6b1548b4668e8271dbe9c&sot=b&sdt=b&sl=106&s=TITLE-ABS-KEY%28%22Ambulance%22+AND+%22Prehospital%22+AND+%22Intensive+Care%22+AND+%22Unit%22+AND+%22Trauma%22+AND+%22Treatment.%22%29&origin=searchbasic&editSaveSearch=&yearFrom=Before+1960&yearTo=Present&sessionSearchId=9b329ac8a4f6b1548b4668e8271dbe9c&limit=10

**CINHAL n = 5:** “Ambulance” AND “Prehospital” AND “Intensive Care” AND “Unit”

https://web-p-ebscohost-com.ezproxy.ub.gu.se/ehost/resultsadvanced?vid=5&sid=face20f0-d264-41ed-874f-ed8b2248af31%40redis&bquery=%22Ambulance%22+AND+%22Prehospital%22+AND+%22Intensive+Care%22+AND+%22Unit%22&bdata=JmRiPWM4aCZ0eXBlPTEmc2VhcmNoTW9kZT1BbmQmc2l0ZT1laG9zdC1saXZl

**WOS n = 8:** “Ambulance” AND “Prehospital” AND “Intensive Care” AND “Unit” AND “Trauma” AND “Treatment”

https://www-webofscience-com.ezproxy.ub.gu.se/wos/woscc/summary/57a9afa0-d792-439d-937c-27d57967daaf-a128a023/relevance/1

**Total number: 71**

**<u>Second Round</u>**

**Duplicates: 4 papers**

**Irrelevant: 25 papers**

**To the next round: 42 papers**

**<u>Third Round</u>**

**<u>Search Results: Prehospital Intensive Care Unit</u>**

**Search result: 42 articles**

**Removed by consensus: 29**

**Primary selected: 7**

**Primary conflict: 6**

**Selected**

1. Apnoeic oxygenation for emergency anaesthesia of pre-hospital trauma patients
2. Efficacy of pre-hospital rapid sequence intubation in paediatric traumatic brain injury: A 9-year observational study
3. Prehospital transfusion of red cell concentrates in a paramedic-staffed helicopter emergency medical service.
4. Termination of prehospital resuscitative efforts: a study of documentation on ethical considerations at the scene
5. Outcome following physician supervised prehospital resuscitation: a retrospective study
6. Statement of severe trauma management in France; teachings of the FIRST study
7. Prehospital activation of a coordinated multidisciplinary hospital response in preparation for patients with severe hemorrhage: A statewide data linkage study of the New South Wales "Code Crimson" pathway

**Conflict (2 yes, 2 no), all accepted for review**

1. The Great Belt train accident: the emergency medical services response
2. Epidemiology of trauma patients attended by ambulance paramedics in Perth, Western Australia
3. A Paramedic-Staffed helicopter emergency medical service's response to winch missions in Victoria, Australia
4. Airway management in unconscious non-trauma patients
5. Impact of helicopter emergency medical service in traumatized patients: Which patient benefits most?
6. A case of blunt traumatic cardiac tamponade successfully treated by out-of-hospital pericardial drainage in a "doctor-helicopter" ambulance staffed by skilled emergency physicians.

Studies

| Study | Titles | Authors |
|---|---|---|
| 1 | Impact of Helicopter Emergency Medical Service in Traumatized Patients: Which Patient Benefits Most? | Andruszkow, H., Schweigkofler, U., Lefering, R., Frey, M., Horst, K., Pfeifer, R., … & Hildebrand, F. (2016). Impact of helicopter emergency medical service in traumatized patients: which patient benefits most?. PLoS One, 11(1), e0146897. |
| 2 | Prehospital activation of a coordinated multidisciplinary hospital response in preparation for patients with severe hemorrhage: A statewide data linkage study of the New South Wales "Code Crimson" pathway | Partyka, C., Miller, M., Johnson, T., Burns, B., Fogg, T., Sarrami, P., … & Dinh, M. (2022). Prehospital activation of a coordinated multidisciplinary hospital response in preparation for patients with severe hemorrhage: A statewide data linkage study of the New South Wales "Code Crimson" pathway. Journal of Trauma and Acute Care Surgery, 93(4), 521–529. |
| 3 | The Great Belt train accident: the emergency medical services response | Hansen, P. M., Jepsen, S. B., Mikkelsen, S., & Rehn, M. (2021). The Great Belt train accident: the emergency medical services response. Scandinavian journal of trauma, resuscitation and emergency medicine, 29(1), 1–14. |
| 4 | Apnoeic oxygenation for emergency anaesthesia of pre-hospital trauma patients | Crewdson, K., Heywoth, A., Rehn, M., Sadek, S., & Lockey, D. (2021). Apnoeic oxygenation for emergency anaesthesia of pre-hospital trauma patients. Scandinavian Journal of Trauma, Resuscitation and Emergency Medicine, 29(1), 1–7. |
| 5 | Efficacy of pre-hospital rapid sequence intubation in paediatric traumatic brain injury: A 9-year observational study | Heschl, S., Meadley, B., Andrew, E., Butt, W., Bernard, S., & Smith, K. (2018). Efficacy of pre-hospital rapid sequence intubation in paediatric traumatic brain injury: a 9-year observational study. Injury, 49(5), 916–920. |
| 6 | Epidemiology of trauma patients attended by ambulance paramedics in Perth, Western Australia: | Brown, E., Williams, T. A., Tohira, H., Bailey, P., & Finn, J. (2018). Epidemiology of trauma patients attended by ambulance paramedics in Perth, Western Australia. Emergency Medicine Australasia, 30(6), 827–833. |

| | | |
|---|---|---|
| 7 | Termination of prehospital resuscitative efforts: a study of documentation on ethical considerations at the scene | Mikkelsen, S., Schaffalitzky de Muckadell, C., Binderup, L. G., Lossius, H. M., Toft, P., & Lassen, A. T. (2017). Termination of prehospital resuscitative efforts: a study of documentation on ethical considerations at the scene. Scandinavian Journal of Trauma, Resuscitation and Emergency Medicine, 25(1), 1–9. |
| 8 | A Paramedic-staffed Helicopter Emergency Medical Service's Response to Winch Missions in Victoria, Australia | Meadley, B., Heschl, S., Andrew, E., De Wit, A., Bernard, S. A., & Smith, K. (2016). A paramedic-staffed helicopter emergency medical service's response to winch missions in Victoria, Australia. Prehospital Emergency Care, 20(1), 106–110. |
| 9 | Prehospital transfusion of red cell concentrates in a paramedic-staffed helicopter emergency medical service: RCC ON HEMS | Heschl, S., Andrew, E., de Wit, A., Bernard, S., Kennedy, M., Smith, K., & Study Investigators. (2018b). Prehospital transfusion of red cell concentrates in a paramedic-staffed helicopter emergency medical service. Emergency Medicine Australasia, 30(2), 236–241. |
| 10 | Outcome following physician supervised prehospital resuscitation: a retrospective study | Mikkelsen, S., Krüger, A. J., Zwisler, S. T., & Brøchner, A. C. (2015). Outcome following physician supervised prehospital resuscitation: a retrospective study. BMJ open, 5(1), e006167. |
| 11 | Statement of severe trauma management in France; teachings of the FIRST study | Tissier, C., Bonithon-Kopp, C., Freysz, M., & French Intensive care Recorded in Severe Trauma (FIRST) study group. (2013, July). Statement of severe trauma management in France; teachings of the FIRST study. In Annales françaises d'anesthésie et de réanimation (Vol. 32, No. 7–8, pp. 465–471). Elsevier Masson. |
| 12 | Airway management in unconscious non-trauma patients | Nielsen, K., Hansen, C. M., & Rasmussen, L. S. (2012). Airway management in unconscious non-trauma patients. Emergency Medicine Journal, 29(11), 887–889. |
| 13 | A Case of Blunt Traumatic Cardiac Tamponade Successfully Treated by Out-of-hospital Pericardial Drainage in a "Doctor-helicopter" Ambulance Staffed by Skilled Emergency Physicians | Otsuka, H., Sato, T., Morita, S., Nakagawa, Y., & Inokuchi, S. (2016). A case of blunt traumatic cardiac tamponade successfully treated by out-of-hospital pericardial drainage in a" doctor-helicopter" ambulance staffed by skilled emergency physicians. Tokai J Exp Clin Med, 41(1), 1–3. |

Summary

Codes and themes

Platform (Violet)

Personnel (Indigo) training (Indigo and underline)

Population (Blue)

Equipments (Green)

Procedures (Yellow)-Interventions/administration of medicines/decision making

Risk (Orange)

Dispatch criteria (Red)

Outcome (yellow highlight)

System organization (green highlight)

| | |
|---|---|
| Outcome following physician supervised prehospital resuscitation: a retrospective study Søren Mikkelsen, 1,2 Andreas J Krüger, 3 Stine T Zwisler, 4 Anne C Brøchner 2, Site—Mobile Emergency Care Unit (MECU) in Odense, Denmark. Time period—1 May 2006 to 31 December 2012 | Anesthesiologist-manned mobile emergency care unit results in a large number of patients receiving prehospital life-saving treatment exceeding the competences of the EMTs or PMs.<br>Prehospital physicians possess some additional critical care competencies that are potentially life-saving but are required infrequently and can carry significant risks. Approximately half of the patients that survive the incident are discharged to their own homes without major or even moderate sequelae. |
| A PARAMEDIC-STAFFED HELICOPTER EMERGENCY MEDICAL SERVICE'S RESPONSE TO WINCH MISSIONS IN VICTORIA, AUSTRALIA. Ben Meadley, BAppSci, GradDip EmergHlth, GradCert EmergHlth, Stefan Heschl, MD, Emily Andrew, BBiomedSc, Anthony de Wit, BHlthSci, GradDip EmergHlth, GradCert, EmergHlth, Stephen A. | The objective of this study was to define the characteristics of winch missions undertaken by Intensive Care Flight Paramedics (ICFPs) in Victoria, Australia, with a focus on extraction methods and clinical care delivered at the scene.<br>Out of 5003 missions in the study period, 125 were identified as winch operations.<br>Patients were predominantly male (78.4%) and had a mean age of 38 years (± 17.6). A total of 109 (87.2%) patients were identified as experiencing trauma with a mean Revised Trauma Score of 7.5288, and isolated limb fractures were the most frequently encountered injury. Falls and vehicle-related trauma were the most common mechanisms of injury. The total median scene duration was 49 min (IQR 23–91). Sixty-three patients (50.4%) were extracted using a stretcher, 45 (36.0%) using a hypothermic strop, and 6 (4.8%) using a normal rescue strop. Eleven patients (8.8%) were not winched |

| | |
|---|---|
| Bernard, MBBS, MD, Karen Smith, BSc, PhD<br>Site—Intensive Care Flight Paramedics (ICFP) in Victoria, Australia.<br>Time period—November 2010 and March 2014. | to the helicopter. Vascular access (38.4%), analgesia (44.0%), and anti-emetic administration (28.8%) were the most frequent clinical interventions.<br>Winch-equipped HEMS deliver highly skilled clinicians directly to patients who could otherwise remain untreated. Winching allows critical clinical interventions to be performed and the patient to be rapidly extracted from the scene. The proposed benefits of winch operations must be weighed against the risk of injury and/or fatality to both crew and patient. Training for and maintaining currency in winch operations, in accordance with the Civil Aviation Safety Authority (CASA) and other legal requirements, incurs a significant financial and operational burden. Fatalities have occurred during winching operations in Australian HEMS operations in recent years, emphasizing the importance of appropriate training and safety measures. |
| The Great Belt train accident: the emergency medical services response Peter Martin Hansen 1,2,8, Søren Bruun Jepsen 3, 3,4,5 and Marius Rehn 6,7,8<br>Site—mobile emergency care units (MECU) stafed by specialists in anaesthesiology with sub-specialization in prehospital critical care. A supplementary physician-manned helicopter EMS (HEMS) Denmark<br>Time period—On 2 January 2019 | The EMS in Denmark is a three-tiered system consisting of ambulances manned by a combination of emergency medical technicians (EMTs) with basic, intermediate, or paramedic levels of training. The Danish EMS also includes rapid response units manned by paramedics and mobile emergency care units (MECUs) staffed by specialists in anesthesia with a sub-specialization in prehospital critical care. A supplementary physician-manned helicopter EMS (HEMS) is also available. Approximately 300 ambulances, 26 MECUs, and 4 HEMS helicopters are available in the Danish EMS.<br>On the tactical level, a Joint Incident Command composed of Police, Fire and Rescue, and Health is responsible for the overall incident command. An ambulance scene commander is responsible for the coordination of the EMS units. The Casualty Clearing Station Officer performs secondary triage, coordinates treatment and allocation of patients with the EMCC physician, and refers to the Medical Incident Commander in the Joint Incident Command.<br>The Great Belt train accident prompted a massive EMS response and involved responders from multiple authorities and private and state contractors. The incident turned out to be the most severe MI in Denmark in thirty years. We aim to describe the immediate prehospital EMS response to the Great Belt Train Accident and evaluate adherence to guidelines to identify areas of improvement for future MI management. This is a retrospective and observational study of the EMS activity on January 2nd, 2019 following the Great Belt train accident. The epidemiological assessment of this MI adheres to CONFIDE (CONsensus guidelines on Reports of Field Observations in Disasters and Emergencies). |
| Efficacy of pre-hospital rapid sequence intubation in paediatric traumatic brain injury: A 9-year observational study<br>Stefan Heschla,b, Ben Meadleyb,c Emily Andrewb,c Warwick Buttd Stephen Bernardb,c,eKaren Smithb,c,f<br>Site—emergency medical service (EMS), Ambulance Victoria,<br>Time period—1 January 2005 and 31 December 2013 | Prehospital RSI in pediatric patients with TBI can safely be performed by highly trained paramedics.<br>Overall, we observed more favorable long-term outcomes in patients who received prehospital intubation than those who did not.<br>We aimed to describe mortality and functional outcomes after six months in children with TBI who either underwent prehospital RSI by a HEMS paramedic or received no intubation. |
| Epidemiology of trauma patients attended by ambulance paramedics in Perth, Western Australia<br>Site St John Ambulance Western Australia (SJA-WA) paramedics in greater metropolitan Perth<br>Time period—2013 and 2016 | The epidemiology of trauma in adult patients attended by ambulance paramedics in Perth, Western Australia (SJA-WA is a single-tier ambulance service and the sole provider of emergency road ambulances in Perth).<br>A retrospective cohort study of trauma patients aged ≥ 16 years attended by St John Ambulance Western Australia (SJA-WA) paramedics in greater metropolitan Perth between 2013 and 2016 found that the majority of patients had low-acuity injuries, with fewer than 3% having high acuity. Advanced life support such as endotracheal intubation, surgical cricothyrotomy, or needle thoracentesis was performed in less than 1% of patients.<br>Focusing research, training, and resources solely on high-acuity patients will not cater to the needs of the majority of patients. |
| Impact of Helicopter Emergency Medical Service in Traumatized Patients: Which Patient Benefits Most?<br>Site—physician staffed helicopter (HEMS) or physician staffed ground emergency medical services (GEMS) Germany<br>Time period—Date of admission from January 2002 until December 2012 | The purpose of the current investigation was to examine whether age, gender, mode of injury, or injury severity could be used to determine specific trauma patient populations who might benefit explicitly from HEMS rescue.<br>HEMS patients were more severely injured compared to GEMS patients (ISS: HEMS 24.8 ± 13.5 vs. GEMS 21.7 ± 18.0) and more frequently suffered traumatic shock (SBP sys < 90 mmHg: HEMS 18.3% vs. GEMS 14.8%). However, logistic regression analysis revealed that HEMS rescues resulted in an overall survival benefit compared to GEMS.<br>Six different procedures of on-scene treatment were documented in the TR-DGU and analyzed in order to evaluate potential differences in management between HEMS and GEMS. These procedures were intubation, insertion of a chest tube, application of vasopressors or sedatives, volume infusion, |

| | |
|---|---|
| | and cardio-pulmonary resuscitation (CPR). HEMS and GEMS physicians are trained standardly in ATLS (Advanced Trauma Life Support) and PHTLS (Prehospital Trauma Life Support) in Germany. Focusing on special subgroups, middle-aged and older patients, low-energy trauma, and minor-severity injuries had the highest survival benefit when rescued by HEMS. Elderly patients aged 55 years and older revealed the strongest survival benefit.<br>Further studies are required to determine the potential reasons for this benefit. |
| Statement of severe trauma management in France; teachings of the<br>FIRST study<br>E´tat des lieux de la prise en charge du traumatise´ grave en France; enseignements<br>de l'e´tude FIRST<br>Site—Mobile Emergency Care Unit or SMUR (Service Mobile d'Urgence et de Re´animation) France<br>Time period—1 December 2004 to 30 April 2007. | The first study was based on a multicenter prospective cohort aged 18 or over with severe exclusive blunt trauma requiring admission to university hospital care units within the first 72 h and/or managed by the Medical-Staffed Emergency Mobile Unit (SMUR). Patients who benefited from medical prehospital management were globally more severely injured than those who received basic life support care from fire brigades. Therefore, they received more aggressive treatment in the prehospital setting, and the median time for their hospital admission was lengthened. However, their 30-day mortality was significantly reduced. The probability of death was also decreased when casualties were transported by SMUR helicopter directly to the university hospital. In the in-hospital setting, the performance of whole-body computed tomography (CT) was associated with a significant reduction in mortality risk compared with selective CT.<br>Patients transported by helicopter (heli-SMUR patients) were presumed to be more severely injured. However, no differences in ISS were noted between the two groups. Hypotension and severe spinal injuries were more frequent in heli-SMUR patients than in ground-SMUR patients. The median time to hospital admission was higher for heli-SMUR patients than for ground-SMUR patients (2.3 h vs. 1.8 h, respectively). In addition, in a prehospital setting, heli-SMUR patients received more aggressive treatment than ground-SMUR patients in terms of endotracheal intubation, crystalloid fluid loading, catecholamine administration, and blood product transfusion. Finally, the risk of death before ICU discharge (within 30 days) was significantly lower for heli-SMUR patients than for ground-SMUR patients after adjustment of the initial status of ISS and of overall surgical procedures. |
| Prehospital activation of a coordinated multidisciplinary hospital response in preparation for patients with severe hemorrhage: statewide data linkage study of the New South Wales "Code Crimson" pathway<br>Site—NSW Ambulance (NSWA) (Avinet Adelaide, South Australia)<br>Time period—between 2017 and 2019<br>Data source—data-linkage study combined prehospital databases with a trauma registry<br>Study population-patients with an Injury Severity Score greater<br>than 12 between | Prehospital medical teams can streamline access to massive transfusions and definitive hemorrhage control by alerting in-hospital trauma teams of suspected life-threatening bleeding in unstable patients. This study reports the initial experience of an Australian "Code Crimson" (CC) pathway facilitating early multidisciplinary care for these patients.<br>There was a greater degree of hemodynamic instability in the CC-activated group, with a higher shock index than in missed, unmatched patients. Code Crimson-activated patients had more multisystem trauma (80%), especially thoracic trauma (including hemopneumothorax, multiple rib fractures, and pulmonary contusion) and femoral fractures, compared with the missed CC patients. The latter more often had a significant traumatic brain injury as well as single organ injuries (41% and 40%). The median shock index was greater than 1 for the CC-activated patients and less than 1 for the missed CC patients.<br>The CC-activated group received a larger number of PH interventions; 60 of 72 (84%) were intubated, 39 of 72 (54%) had chest decompression, 30 of 72 (42%) had a positive eFAST, and 71 of 72 (99%) received blood products consistent with CC criteria. |
| Apnoeic oxygenation for emergency anaesthesia of pre-hospital trauma patients<br>Site—two UK pre-hospital services who underwent PHEA were included in the study<br>two physician-paramedic manned pre-hospital services. Doctor–paramedic teams are delivered by helicopter and fast response cars. Flight paramedics in the ambulance control room dispatch the services and specific dispatch criteria target critically ill or injured patients<br>Time period—February 2016. | Rapid and effective airway management is a priority for major trauma patients. Advanced airway interventions are necessary for a small subgroup of severely injured patients, in whom basic airway interventions are inadequate to maintain oxygenation and ventilation.<br>Prehospital emergency anesthesia (PHEA) is required to facilitate tracheal intubation and ventilation. Adverse events associated with intubation occur more frequently during emergency airway management, particularly when repeated attempts at laryngoscopy are required.<br>This study aims to investigate whether the introduction of apneic oxygenation would reduce the frequency of desaturation in a population of trauma patients undergoing PHEA.<br>This is the first reported prospective study of the use of apneic oxygenation during PHEA in trauma patients.<br>In this study, apneic oxygenation did not influence peri-intubation oxygen saturations, but it did reduce the frequency and duration of hypoxia in the post-intubation period. Given that apneic oxygenation is a simple, low-cost intervention with a low complication rate and that hypoxia can be detrimental to outcome, the application of nasal cannulas during the drug-induced phase of emergency intubation may benefit a subset of patients undergoing emergency anesthesia. |
| A Case of Blunt Traumatic Cardiac Tamponade Successfully Treated by Out-of-hospital Pericardial Drainage in a "Doctor-helicopter" Ambulance | We report a 55-year-old man who relapsed into a state of shock in an ambulance before arriving at our critical care center after a fall injury. The diagnosis of cardiac tamponade was made by portable ultrasound, and immediate pericardiocentesis and drainage were performed at the heliport. |

| | |
|---|---|
| Staffed by Skilled Emergency Physicians<br>Site—the “doctor-helicopter” ambulance transportation system<br>Time period—2015 | The procedure was being performed in the narrow confines of the inside of the ambulance, where stretcher manipulation was difficult. Ultrasound was used to search for the optimum point in the supine position, and the left 5th intercostal space was selected.<br>This case illustrates that such patients can be treated reliably by pericardial drainage performed by skilled emergency physicians in the field by making use of the “doctor-helicopter” ambulance transportation system. |
| Airway management in unconscious non-trauma patients<br>Site—the MECU database in Copenhagen, Denmark.<br>Time period—during 2006 | The aim of this study was to describe the authors’ experience with airway management in unconscious non-trauma patients in a prehospital setting with a physician-manned Mobile Emergency Care Unit (MECU). The main focus of the study was on the need for subsequent tracheal intubation during hospitalization after initial treatment.<br>A total of 557 unconscious non-trauma patients were examined, and 129 patients (23%) were tracheally intubated by the MECU physician before or during transport to the hospital. Intubation was performed in most patients with cardiac arrest, severe stroke, or respiratory failure. |
| Termination of prehospital resuscitative efforts a study of documentation on ethical considerations at the scene<br>Site—Danish prehospital setting Mobile Emergency Care Unit in Odense<br>Time period—2010 to 2014. | The study is based on discharge summaries of all patients subjected to crucial life-and-death decisions by the Mobile Emergency Care Unit in Odense in the years 2010 to 2014. The medical records with possible documentation of ethical issues were independently reviewed by two philosophers in order to identify explicit ethical or philosophical considerations pertaining to the decision to resuscitate or not. In a total of 62 patients, 85 specific ethical issues related to resuscitation were documented. The expressions of the ethical considerations were generally vague or unclear and almost exclusively concerned the interests of the patient and not the relatives. |
| Prehospital transfusion of red cell concentrates in paramedic-staffed helicopter emergency medical service<br>Site—paramedic-staffed helicopter emergency medical system in<br>Victoria, Australia.<br>Time period—July 2011 and December 2015 | The study aimed to describe the implementation and initial experience of Red Cell Concentrate administration in a paramedic-staffed helicopter emergency medical service (HEMS) in Victoria, Australia.<br>A total of 150 patients received prehospital RCCs, of whom 136 had suffered trauma. The majority of these patients were male (66.7%) and were involved in a car accident (62.5%). Most (97.4%) patients had an Injury Severity Score ≥ 12. There were improvements in median systolic blood pressure (80 mmHg vs. 94 mmHg, $p < 0.001$) and shock index (1.50 vs. 1.23, $p < 0.001$) between the time of consultation and arrival at the hospital. Overall, mortality for trauma patients was 37.7%. There were no transfusion-related complications identified. |
