# Supplementary material for: The Use of Prehospital Intensive Care Units in Emergencies—A Scoping Review
Source: Healthcare (Basel). 2023 Nov 2;11(21):2892. doi: 10.3390/healthcare11212892 (PMC10647734; doi:10.3390/healthcare11212892)
Supplement: Supplementary file 1 [file healthcare-11-02892-s001.zip › Supplementary File S2.pdf]

healthcare

MDPI

Supplementary File S2

Biographic Details	Setting/Context	Population	Study Design	Interventions/Groups	Outcome(s) Measured	PICU Characteristics*	Main Findings	Comments
Andruszkow H (2016) [1]. Impact of Helicopter Emergency Medical Service in Traumatized Patients: Which Patient Benefits Most? PLoS ONE	A regional trauma system registry (prehospital (HEMS and GEMS)) and hospital (trauma centers) in Germany	52,281 trauma patients	Retrospective cohort	HEMS vs. GEMS in prehospital trauma care (intervention); survival, hospital stay, and complications (groups)	Survival, hospital stay, and complications	Physicians working on HEMS and GEMS are trained in ATLS and PHTLS, on-scene procedures, and the prehospital management of trauma patients in general. The authors did not mention the PICU size, capacity, type of equipment, protocols, and practice but mentioned monitoring of the HEMS/GEMS operation mission duration and the Glasgow Coma Scale as standard in an electronic database. Patients were transported to a Level I or II trauma center.	The study included 52,281 trauma patients, of whom 68.8% were rescued by GEMS and 31.2% by HEMS. HEMS patients were more severely injured and more likely to suffer traumatic shock than GEMS patients. Trauma patients were primarily treated by HEMS or GEMS, and documentation was performed using a trauma register. Prolonged overall mission duration (HEMS: 76.6 ± 28.2 min vs. GEMS: 60.7 ± 26.7 min) and on-scene time (HEMS: 37.0 ± 20.4 min vs. GEMS: 28.0 ± 16.0 min) were recorded. Logistic regression analysis revealed that HEMS rescues resulted in an overall survival benefit.	HEMS rescues resulted in an overall survival benefit compared to GEMS.
Partyka C (2016) [2]. Prehospital activation of a coordinated multidisciplinary	Multiple data registries, including prehospital, hospital, and trauma registries, for CC activation in NSW, Australia	226 prehospital patients with and without in-hospital links, patients	Retrospective data linkage	CC activation vs. non-CC activation for trauma patients with severe hemorrhage (intervention); hemorrhage	Hemorrhage control, mortality, and hospital and ICU stay	HEMS, fixed-wing planes, and GEMS were staffed by physicians and paramedics trained in emergency medicine, anesthesia, or intensive care. The authors mentioned standard	Most CC patients were young men with blunt trauma, in need of more prehospital interventions than non-CC patients. CC patients had more multisystem trauma, especially complex	The initiation of a statewide CC process was highly specific to the need for hemorrhage control intervention in hospitals, but further studies are

hospital response in preparation for patients with severe hemorrhage: A statewide data linkage study of the New South Wales "Code Crimson" pathway Journal of Trauma and Acute Care Surgery	with inpatient treatment consistent with Code Crimson without being activated, and potentially missed patients in Australia	control, mortality, and hospital and ICU stay (groups)		equipment for prehospital care, hemorrhage control, blood product sets, and eFAST. The authors did not mention PICU size or capacity but rather the protocol for CC activation and standard trauma care guidelines for treatment. Paramedics communicate with the dispatch center to activate CC. Documentation through the quality assurance database. On completion of each patient contact, physicians from each service complete a report in the comprehensive quality assurance database that includes patient demographics, injury details, treatments/interventions, blood products, medication delivery, and ED arrival time.	thoracic injuries (80%), while missed CC patients more often had single organ injuries (59%). CC patients needed fewer hemorrhage control procedures (60% vs. 86%). Lower mortality was seen in CC patients despite greater hospital and intensive care unit lengths of stay. Prehospital CC activation was highly specific to the need for hemorrhage control intervention in the hospital. CC-activated patients had lower mortality. Patients received hemorrhage control interventions in the prehospital setting. Around 60% of CC-activated patients needed hemorrhage control interventions, compared to 86% of matched missed CC patients and 71% of unmatched missed CC patients. Moreover, the CC-activated group received a larger number of prehospital interventions; 84% were intubated, 54% had chest decompression, 42% had a positive eFAST, and 99% received blood products	needed to improve the sensitivity of prehospital activation. The patterns and types of injuries in trauma patients help improve immediate care and interventional procedures. CC-activated patients had more multisystem trauma but lower mortality. These results help to improve CC activation and find subgroups that may benefit from it.

							consistent with CC criteria.	
M. Hansen P (2016) [3]. The Great Belt train accident: the emergency medical services response Scandinavian Journal of Trauma, Resuscitation and Emergency Medicine	Incident command system (multiple databases including prehospital, hospital, media reports, and others) in Denmark	Officials, EMS providers involved in the incident, records of the 15 patients involved in the incidents, incident management, and media report records	Descriptive case report	EMS response to the train accident; no comparison (intervention); casualties, resources, actions, triage, transport, destinations, capacities, challenges, barriers, and lessons learned (groups)	Casualties, resources, actions, triage, transport, destinations, capacities, challenges, barriers, and lessons learned	HEMS (and SAR helicopters), MECU, RRU, and GEMS are staffed by anesthesiologists with a sub-specialization in prehospital critical care (MECU), paramedics (RRU), and EMTs (ambulances). Three MECUs, two HEMS, and one SAR helicopter took part in this MI (of a total of 28 MECUs), 4 HEMS helicopters, and 3 SAR helicopters available in the Danish EMS: The capacity is not reported. Danish guidelines for interdisciplinary MI management guidelines and a criteria-based dispatch protocol are stated. The EMS uses a HEALTH channel and switches to the nationwide emergency radio network (SINE) channel during the MI. The prehospital electronic patient record system in Denmark is available for EMS units involved in an MI, enabling the registration of the injuries and the triage levels of the	Radio communication was partly compromised, with 38.9% of the radio shifts not according to the planned radio grid. Access to the incident site was challenging and delayed due to traffic congestion and safety issues. Triage was relevant, but at the physicians' discretion. EMS did the first and secondary triage, evacuation, and patient transportation. As no patients needed transport over longer distances and as the nature of the injuries in the patients did not require extensive medical treatment at the scene, the HEMS helicopters were canceled on-scene.	Despite the communication challenges, safety concerns, and difficult access to the incident site, the incident was successfully managed due to solid MI management concepts, substantial training, and adherence to national guidelines. Lessons learned from this incident led to improvements in prehospital units and MI certification, while findings also suggested the need to improve EMS safety issues and further investigations regarding PICU roles in MI.

						patients at the scene of the incident.		
Crewdson K (2016) [4]. Apnoeic oxygenation for emergency anesthesia of pre-hospital trauma patients Scandinavian Journal of Trauma, Resuscitation and Emergency Medicine	Prehospital and hospital settings in the UK	725 patients receiving standard care vs. those receiving additional oxygen by nasal prongs	Prospective before-after	Additional apneic oxygen by nasal prongs vs. standard care for trauma patients who underwent an intervention; oxygen saturation, hypoxia, bradycardia, hypotension, intubation success rate, and complications (groups)	Oxygen saturation, hypoxia, bradycardia, hypotension, intubation success rate, and complications	HEMS was staffed by emergency physicians, anesthesiologists, and paramedics. Anesthesiologists and paramedics receive a training package before the introduction of any intervention. Capnography and patient monitors were used to assess the progress; however, equipment to administer oxygen could also have been used. The authors did not mention PICU size or capacity. The stated clinical treatment was protocol-led, and all clinicians working within the services were fully aware of it. Documentation was performed using continuous monitoring and data recording protocols.	Apneic oxygenation had become embedded into routine practice. Patients received emergency intubation in the prehospital setting. Apneic oxygenation did not influence peri-intubation oxygen saturations, but it did reduce the frequency and duration of hypoxia in the post-intubation period. There was a statistically significant benefit from apneic oxygenation in reducing the frequency of peri-intubation hypoxia ($SpO2 < = 90\%$) for patients with initial $SpO2 > 95\%$. The other significant benefit was observed in the recovery phase for patients with severe hypoxia before intubation.	Hypoxia is relatively frequent during emergency airway management. Apneic oxygenation is a low-cost intervention with low complication rates and is recommended during emergency anesthesia. It does not influence peri-intubation O_2 saturations but reduces the frequency and duration of hypoxia in the post-intubation period. Randomized, controlled studies are needed.
Heschl S (2016) [5]. Efficacy of pre-hospital rapid sequence intubation in paediatric traumatic	Prehospital and hospital phases based on a trauma registry in Victoria, Australia	106 children in Victoria, Australia, with suspected traumatic brain injury (TBI)	Retrospective study	Prehospital RSI by ICP vs. no intubation for children with suspected TBI (intervention); hospital mortality and functional outcomes (groups)	Hospital mortality and functional outcomes	HEMS is staffed by ICPs, aircrewmen, and pilots. ICPs are trained and authorized to perform ETI on adult and pediatric patients in a variety of different scenarios, such as respiratory arrest,	Patients received prehospital rapid sequence intubation (RSI) by trained ICPs. The intubation success rate was 99% (86/87), with a first-pass success rate of 93% (81/87). In total, 67% of patients (n = 41)	Prehospital RSI in pediatric patients with TBI can safely be performed by highly trained paramedics, despite the existing controversies about prehospital airway

brain injury: A 9-year observationa l study Injury	cardiac arrest, and impaired consciousness. There were five HEMS available across the region. The capacity was not stated. HEMS used the injury severity assessment (ISS) and measured impairment using GCS. The stated injury classification protocol was used based on criteria for minor and major trauma (defined as an ISS > 15). Patients were transported and transferred to the nearby Royal Children's Hospital in Melbourne.	receiving RSI had a favorable functional outcome, compared with 54% of non-intubated patients (n = 7) (*p* = 0.36). In the 75 children with major trauma, prehospital RSI was associated with a significant decrease in length of hospital stay (523 h vs. 1939 h, *p* = 0.03). In the 53 children in this subgroup with available six-month data, the difference in favorable functional outcome increased to 66% (n = 31) vs. 17% (n = 1) (*p* = 0.06). There was no significant difference in overall mortality or functional outcome between children with TBI who received RSI and those who did not, though RSI was associated with a decreased length of hospital stay in major trauma groups. Prehospital RSI in pediatric patients with TBI can safely be performed by highly trained paramedics. Overall, more favorable long-term outcomes were seen in patients who received prehospital intubation than those who did not. Intubation before	management of pediatric patients with TBI. This study did not aim to detect a significant difference in long-term outcomes between patients who received prehospital intubation and those who did not. Current guidelines suggest no evidence to support the superiority of out-of-hospital ETI over bag valve mask ventilation in pediatric patients with traumatic brain injury (TBI). Further studies are needed to verify the pros and cons of prehospital RSI in children.

							transport might be beneficial for major trauma patients.	
Brown E (2016) [6]. Epidemiology of trauma patients attended by ambulance paramedics in Perth, Western Australia Emergency Medicine Australasia	Prehospital phase based on the St. John Ambulance Registry in Perth, Australia	97,724 trauma patients aged 16 years or older were attended by St. John Ambulance Western Australia (SJA WA) paramedics in greater metropolitan Perth between 2013 and 2016	Retrospective cohort	Immediate deaths, early deaths, late deaths, and survivors of adult trauma patients attended by paramedics (intervention); incidence and 30-day mortality rates (groups)	Incidence and 30-day mortality rates	GEMS is staffed by paramedics with advanced life support skills, e.g., ETI (non-rapid sequence), medication administration through vascular access, and manual defibrillation. Most of the patients (75.8%) attended by paramedics had low acuity. The author suggested that the current training focusing solely on high-acuity patients will underestimate the true burden of injury, not catering to the needs of most patients. They report that MECU can perform advanced life support procedures, and thus ambulances carry equipment to perform procedures such as ETI, vascular access, and manual defibrillation. The PICU size and capacity were not mentioned. Clinical Practice Guidelines and protocol-based dispatching using a computer-aided	The trauma incidence rate increased over time in Perth, Western Australia. Most patients had low-acuity injuries. Patients received advanced airway management, vascular access, medication administration, and hemorrhage control interventions in the prehospital setting. The administration of analgesic drugs was the most common intervention performed by paramedics, with 40.1% of patients receiving at least one analgesic agent, most often intravenous fentanyl (24.7%) followed by the inhaled anesthetic methoxyflurane (15.1%). Almost a third of patients had an intravenous catheter inserted (n = 25 060/80 643, 31.1%). Advanced life support such as endotracheal intubation, surgical cricothyrotomy, or needle thoracentesis was performed in less than 1% of patients. Finally, the authors suggested that focusing research,	Motor vehicle accidents contribute to immediate and early deaths, particularly among younger individuals. The findings of this study suggest most trauma patients are not high acuity, but it remained the main focus of trauma research, training, and resource allocation. Therefore, there is a need to tailor research, training, and resources to the needs of most patients.

						dispatch system were stated. Documentation was an electronic patient care record system.	training, and resources solely on high-acuity patients will not cater to the needs of the majority of patients.	
Mikkelsen S (2016) [7]. Termination of prehospital resuscitative efforts: a study of documentation on ethical considerations at the scene Scandinavian Journal of Trauma, Resuscitation and Emergency Medicine	Prehospital Mobile Emergency Care Unit (MECU) registry in Odense, Central Denmark Region, Denmark	1275 patients in a Danish prehospital setting for whom the decision to resuscitate or not was made at the scene	Retrospective analysis of discharge summaries	Ethical considerations and decision-making documentation in life-or-death decisions by anesthesiologists; no comparison (intervention); ethical considerations and decision-making documentation (groups)	Ethical considerations and decision-making documentation	Ground MECU and RRU are staffed by anesthesiologists, paramedics, and EMTs. The authors did not mention PICU size, capacity, or equipment. Stated a criteria-based protocol to dispatch MECU. Documentation is performed in the form of a discharge summary. EMTs communicated with the dispatch center to activate MECU, and a transfer was carried out to a nearby hospital. MECU physicians document patient conditions and treatment in the patient's medical records.	Resuscitation, crucial life and death decisions, and documentation on medical records by the MECU physicians on scene were evaluated in 1275 patients. In 642 of these patients, on-scene resuscitative efforts were initiated. In 633 patients, natural death was allowed without any resuscitative efforts being started. In 36 of the 633 patients not resuscitated (5.9% (4.0–7.8%)), a DNR (Do-Not-Resuscitate) order was presented to the MECU anesthesiologist. Among the 1275 patients in whom the decision to resuscitate or not was made by the MECU physician, only 62 patients (4.9% (3.7–6.2%)) had medical records containing specific ethical or philosophical considerations about the event. All DNR orders were either formulated as written or verbal instructions from the patient, the patient's general practitioner, the	There is a need for the strengthened practice of documenting ethical considerations in prehospital life and death decision-making in the patient's medical records. Moreover, quality assurance interventions and guidelines towards explicit inclusion of certain points in medical records are needed; this may include systematic recording of persons involved in deliberations, notes on ethical considerations, estimations of quality of life, and a summary of other ethical concerns.

							hospital in charge of treatment, or the next-of-kin. Documentation of ethical considerations in prehospital life-and-death decision-making is generally vague or unclear. In most cases where ethical content was found, the ethical considerations led to a decision to terminate treatment. A template should be implemented in the prehospital medical records describing the basis for any ethical decisions, including the patient's end-of-life wishes, estimations of the quality of life before and after the incident, and a summary of other ethical concerns.	
Meadley B (2016) [8]. A Paramedic staffed helicopter emergency medical services response to winch mission in VICTORIA, AUSTRALI A Prehospital	Prehospital phase registry of Air Ambulance Vehicles (AAVs) in Victoria, Australia	125 predominan tly male (78.4%) patients aged 38 years (± 17.6) who were treated during winch missions by Intensive Care Flight Paramedics	Retrospective study	Winch missions by ICFPs; no comparison (intervention); RTSc for trauma patients (groups)	RTSc for trauma patients	HEMS is staffed by an intensive care flight paramedic (ICFP), an aircrewman, and a pilot. The ICFP crew is trained in water rescue techniques and undergoes regular skill reaccreditation. Air crewmen have over 120 h of training in their role as emergency medical assistants. ICFPs staffing HEMS at Air Ambulance Victoria (AAV) undergo	Winch missions were predominantly undertaken in the winter and involved mostly male patients with a mean age of 38 years. Falls and vehicle-related trauma were the most common mechanisms of injury, with isolated limb fractures being the most common injury. Vascular access, analgesia, and anti-emetic administration were the most frequent	Many patients did not need any clinical interventions before the winching process. However, winch missions in AAV-HEMS have potential benefits, including providing access to remote locations, reducing transport times, and improving patient outcomes.

Emergency Care		in Victoria, Australia				extensive education and training. Recertification in water operations occurs annually at a dedicated 1-day exercise and includes specific water rescue technique revision, practice, and an element of swimming to ensure competence. There were five HEMS available in Victoria, including five aircraft (four Bell 412 EP and one Airbus Dauphine N3). The capacity is not stated. There were no explicitly reported protocols, but the authors stated protocol-led winching to the scene, extraction, and transfer of the patients. HEMS provides primary response missions and inter-facility transfers. Documentation was electronic for patient care records.	clinical interventions and were performed in the prehospital setting. Response to the scene was given, and a total median scene duration was 49 min, with similar durations for trauma and search and rescue cases (48 and 51 min, respectively). Medical cases had a median scene time of 76 min; however, due to the small sample size, the difference between groups did not reach statistical significance ($p = 0.63$). The most common access/egress method involved the paramedic being winched into the scene, and the patient then winched out; this occurred in 105 cases (84.0%). In 11 (8.8%) cases, the paramedic was winched into the scene but chose to transport the patient out by road ambulance or another vehicle. A total of 49 (39.2%) patients received no clinical interventions on the scene.	
Heschl S (2016) [9]. Prehospital transfusion of red cell	Prehospital and hospital phases based on a state-wide trauma	150 mostly male patients in Victoria, Australia,	Retrospective study	RCC transfusions by paramedics in a HEMS; no comparison (intervention);	Mortality, RCC usage, blood unit waste rates, transport time, and	HEMS is staffed by an ICFP (intensive care paramedic), an aircrewman, a pilot, and consultant physicians.	Transfusion of red cell concentrates (RCCs) and monitoring of the patients were conducted in the prehospital setting.	Prehospital RCC transfusion is a novel practice with potential benefits, including improved

concentrates in a paramedic-staffed helicopter emergency medical service Emergency Medicine Australasia	registry in Victoria, Australia	who received prehospital red cell concentrates (RCCs)		mortality, RCC usage, blood unit waste rates, transport time, and physician consultation (groups)	physician consultation	The HEMS ICFP undergoes extensive training and has advanced skills for the treatment of critically ill patients compared with road-based paramedics. Their skillset includes, but is not limited to, advanced airway management by rapid sequence intubation (RSI) and cricothyroidotomy. The ICFP consults the responsible physician for approval of RCC transfusions. The authors mentioned that HEMS (in AAVs) carried standard equipment for prehospital care and blood product sets. The authors did not mention PICU size or capacity. They stated standard operating procedures for RCC transfusions. RTSc, initial vital signs, and shock index were used to classify injury severity. While the primary response mission was included in the study, transport and scene time were monitored starting from the first responding scene, then from	Consultation with an ARV physician coordinator was needed before the administration of RCC. The RCC units were provided by local blood banks to the four AAV bases, with a wastage rate of 0.5%. Most paramedic consultations to administer RCC were approved by the attending physician (90%). Six patients received RCCs for non-traumatic conditions that involved massive hemorrhages. There were improvements in median systolic blood pressure and shock index between the time of consultation and arrival at the hospital. There were no transfusion-related complications identified. 150 patients received prehospital RCCs, most of whom were male and involved in a car accident.	hemostasis, reduced mortality and morbidity, and better patient outcomes, despite controversies about the optimal volume and type of intravenous fluid for the treatment of blood loss. Further research is needed to find the optimal use of prehospital resuscitative fluids and their effects on patient outcomes, as well as comparative studies of prehospital RCCs with in-hospital administration to evaluate the effects of prehospital RCCs on patient outcomes.

						departure from the scene to arrival at the hospital. An electronic patient care record, which includes patient and case details and clinical management, is completed by the paramedic after each mission.		
Mikkelsen S (2016) [10]. Outcome following physician supervised prehospital resuscitation : a retrospective study BMJ Open	Prehospital phase (MECU) registry in Odense, Central Denmark Region, Denmark	25,647 patients in Odense, Denmark, who received prehospital 'life-saving treatment' from the Mobile Emergency Care Unit (MECU)	Retrospective study	Prehospital resuscitation by the MECU; no comparison (intervention); the etiology of the life-threatening condition, the level of competence necessary to treat the patient, and the survival rate (groups)	The etiology of the life-threatening condition, the level of competence necessary to treat the patient, and the survival rate	The ground MECU is staffed by an EMT/PM and an anesthesiologist. Anesthesiologists have at least 7 years of postgraduate experience and a minimum of 5 years of training within the specialty. The authors assessed the competencies needed to save a patient, deciding whether they fell within the competencies of the attending physician or emergency medical technician (EMT) or exceeded their competencies. They found specific criteria for lifesaving interventions, highlighting that these competencies are often outside the scope of an EMT's or paramedic's competencies. The authors did not mention PICU size, capacity, or	Prehospital care provided by physician-based emergency services (P-EMS) increases survival and favorable outcomes. Anesthesiologist-administered prehospital therapy increases the level of treatment modalities, leading to increased survival. Specialists in anesthesiology should be applied in the prehospital setting, especially when treating patients with cardiac arrest, patients in need of respiratory support, and trauma patients. 701 patients received prehospital "lifesaving" treatment; of these, 596 were subjected to lifesaving interventions performed by the attending physician; for the remaining 102 patients, the treatment necessary to save the patient's life was	Physician-supervised resuscitation is a common practice in some EMS systems, with potential benefits such as advanced care, reduced mortality and morbidity, and improved patient outcomes. Anesthesiologist-administered prehospital therapy increases the level of treatment modalities, leading to increased survival in the prehospital setting, especially when treating patients with cardiac arrest, patients in need of respiratory support, and trauma patients. Future studies are needed

						equipment but rather stated a protocol-based dispatching system with two different criteria systems for the dispatch process. Patients generally left the scene after treatment. Patient demographics, clinical characteristics, and tentative diagnoses were entered into the MECU database.	administered within the competence of the attending EMT or PM.	in the prehospital setting.
Tissier C (2016) [11]. Statement of severe trauma management in France; teachings of the FIRST study.	Prehospital and hospital settings (HEMS/GEMS and trauma centers) in France	Patients aged 18 or older with severe exclusive blunt trauma requiring admission to a university hospital care unit within the first 72 h and/or managed by a medical-staffed Staffed Emergency Mobile Unit (SMUR) in France	Multi-center prospective cohort	Prehospital medical management by SMUR vs. basic life support care by fire brigades (intervention); 30-day mortality or ICU discharge; mode of transport and level of hospital influence; imaging benefit; triage score performance (groups)	30-day mortality or ICU discharge; mode of transport and level of hospital influence; imaging benefit; triage score performance	The HEMS (SMUR helicopter) and ground ambulance are staffed by emergency physicians. The authors do not report the training of the providers. The authors did not mention PICU size, capacity, or equipment. The treatment provided in prehospital settings was evaluated against existing protocols. Patients were assessed for hemodynamic instability and injury severity. After medical management at the scene, patients were transported to a Level I trauma center. Documentation is submitted in French Intensive Care Recorded	The majority 68.8% (35,974) of the patients were rescued by GEMS, 31.2% (16,307) patients were rescued by HEMS, and patients received various medical management of trauma in the prehospital setting. The findings show that 97% of the 2703 patients received help from prehospital SMUR management, and 7% received BLS from non-medical fire brigades (non-SMUR patients). SMUR management significantly delayed admission to the first hospital and to the ICU compared with non-SMUR patients. The median on-scene and transport times were significantly higher for SMUR patients than for	Medical management in the prehospital setting was beneficial for the survival of trauma patients.

						in Severe Trauma (FIRST). The monitoring of indicators (end-tidal CO_2) was missing in one-half of cases, and regional registries are missing.	non-SMUR patients. The use of vasopressors, blood product transfusions, and endotracheal intubation increased after prehospital medical management. Prehospital medical management was associated with a reduction in 30-day mortality. Transporting casualties by SMUR helicopter directly to the university hospital was associated with a decrease in mortality risk. Whole-body computed tomography (CT) was associated with a significant reduction in mortality risk compared with selective CT.	
Nielsen K (2016) [12]. Airway management in unconscious non-trauma patients Emergency Medicine Journal	Prehospital phase (MECU), based on a registry in Denmark	557 unconscious non-trauma patients in Copenhagen, Denmark	Retrospective study	Airway management by MECU in unconscious non-trauma patients; no comparison (intervention); prehospital and post-admission ETI proportion; diagnosis, outcome, and need for intubation (groups)	Prehospital and post-admission tracheal intubation proportion; diagnosis, outcome, and need for intubation	Ground MECU is staffed by anesthesiologist doctors and advanced life-support providers who are specially trained anesthesiologists. It was not explicitly reported, but the authors mentioned that providers are specially trained in advanced life-support skills. The authors did not mention PICU size, capacity, or equipment. A MECU database as a	Of the 557 unconscious non-trauma patients examined, 129 (23%) were intubated through the trachea by the MECU physician before or during transport to the hospital. Of the remaining 428 patients, 364 (85%) regained consciousness before being transported to the hospital, whereas 64 patients remained unconscious during transport, and 12 (19%) of these were intubated in the emergency	Although most unconscious non-trauma patients did not need tracheal intubation before being transferred to the hospital, prehospital airway management was a challenging task with potential impacts on their survival outcomes. Further research should provide firm recommendations about airway

						registration system exists where prospective registration of patients in the database includes details of information provided by MECU physicians. However, there was inconsistent charting for GCS.	department. Most unconscious non-trauma patients were not intubated in the prehospital setting. 129 patients received endotracheal intubation in a prehospital setting for medical emergencies.	management in unconscious non-trauma patients and the use of GCS.
Otsuka H (2016) [13]. A Case of Blunt Traumatic Cardiac Tamponade Successfully Treated by Out-of-Hospital Pericardial Drainage in a "Doctor-helicopter" Ambulance Staffed by Skilled Emergency Physicians. The Tokai Journal of Experimental and Clinical Medicine	Prehospital and hospital settings in Japan	a 55-year-old patient.	A case report	Prehospital pericardial drainage for a case of cardiac tamponade; comparison NA (intervention); prehospital and hospital course and outcome; complications (groups)	Prehospital and hospital course and outcome; complications	HEMS (doctor-helicopter) is staffed by a doctor-helicopter team including an emergency medicine physician, a certified emergency nurse, and a pilot. HEMS was equipped with Focused Assessment with Sonography for Trauma (FAST), ultrasonography, and a pigtail catheter. The authors did not mention PICU size or capacity. Protocols were not explicitly reported, but radio communication was carried out to prepare for emergency thoracic surgery, while physical examination and FAST were used to continuously assess and monitor the patient in a prehospital setting.	A 55-year-old man was successfully treated for blunt traumatic cardiac tamponade by out-of-hospital pericardial drainage in a "doctor-helicopter" ambulance staffed by skilled emergency physicians. A portable ultrasound was used to diagnose the cardiac tamponade, and immediate pericardiocentesis and drainage were performed at the heliport. The patient was then transported to a critical care center for surgery and made a full recovery after rehabilitation. The doctor-helicopter team performed pericardial aspiration using a pigtail catheter under ultrasonographic guidance in the prehospital setting.	Pericardial drainage was a rare and challenging procedure that had the potential to improve hemodynamics, reduce mortality and morbidity, and improve patient outcomes. Pericardial drainage performed by skilled emergency physicians in the field in "doctor-helicopter" EMS, followed by surgical intervention in a critical care center, has reliably saved a patient with blunt traumatic cardiac tamponade. There is a need for further research to verify the need for this intervention (out-of-hospital pericardial drainage) and the

	procedures used, as well as compare it with other interventions.

* PICU configuration includes: platform (air vs. group), physicians/paramedics, equipment/possible interventions, and EMS systems. HEMS: Helicopter Emergency Medical Service; PH: Prehospital; CC: Code Crimson; EMS: Emergency Medical Services; MI: Myocardial Infraction; TBI: Traumatic Brain Injury; ICFP: Intensive Care Flight Paramedics; RCC: Red Cell Concentrate; PM: Paramedic; GCS: Glasgow Coma Scale; MECU: Mobile Emergency Care Unit; ATLS: Advanced Trauma Life Support; PHTLS: Prehospital Trauma Life Support; EMTs: Emergency Medical Technicians; ICPs: Intensive Care Paramedics; ALS: Advanced Life Support; CPG: Clinical Practice Guidelines; RSI: Rapid Sequence Intubation; ABG: Arterial Blood Gases; Hb: Hemoglobin; EMP: Emergency Medicine Physician; ISS: Injury Severity Score; US: Ultrasound; ETI: Endotracheal Intubation; CRP: Cardiopulmonary Resuscitation; SBP: Systolic Blood Pressure; NR: Not Reported; GEMS: Ground Emergency Medical Services; SMUR: Medical-Staffed Emergency Mobile Unit; YOP: Year of Publication; eFAST: Focused Assessment with Sonography for Trauma; SAR: Search and Rescue.

References

1. Andruszkow, H.; Schweigkofler, U.; Lefering, R.; Frey, M.; Horst, K.; Pfeifer, R.; Beckers, S.K.; Pape, H.C.; Hildebrand, F. Impact of helicopter emergency medical service in traumatized patients: Which patient benefits most? *PLoS ONE* **2016**, *11*, e0146897
2. Partyka, C.; Miller, M.; Johnson, T.; Burns, B.; Fogg, T.; Sarrami, P.; Singh, H.; Dee, K.; Dinh, M. Prehospital activation of a coordinated multidisciplinary hospital response in preparation for patients with severe hemorrhage: A statewide data linkage study of the New South Wales "Code Crimson" pathway. *J. Trauma Acute Care Surg.* **2022**, *93*, 521–529
3. Hansen, P.M.; Jepsen, S.B.; Mikkelsen, S.; Rehn, M. The Great Belt train accident: The emergency medical services response. Scand. J. Trauma Resusc. Emerg. Med. 2021, 29, 140.
4. Crewdson, K.; Heywoth, A.; Rehn, M.; Sadek, S.; Lockey, D. Apnoeic oxygenation for emergency anaesthesia of pre-hospital trauma patients. Scand. J. Trauma Resusc. Emerg. Med. 2021, 29, 10.
5. Heschl, S.; Meadley, B.; Andrew, E.; Butt, W.; Bernard, S.; Smith, K. Efficacy of pre-hospital rapid sequence intubation in paediatric traumatic brain injury: A 9-year observational study. *Injury* **2018**, *49*, 916–920
6. Brown, H.A.; Douglass, K.A.; Ejas, S.; Poovathumparambil, V. Development and Implementation of a Novel Prehospital Care System in the State of Kerala, India. *Prehosp. Disaster. Med.* **2016**, *31*, 663–666. https://doi.org/10.1017/S1049023X16000960.
7. Mikkelsen, S.; Schaffalitzky de Muckadell, C.; Binderup, L.G.; Lossius, H.M.; Toft, P.; Lassen, A.T. Termination of prehospital resuscitative efforts: A study of documentation on ethical considerations at the scene. *Scand. J. Trauma Resusc. Emerg. Med.* **2017**, *25*, 35
8. Meadley, B.; Heschl, S.; Andrew, E.; De Wit, A.; Bernard, S.A.; Smith, K. A paramedic-staffed helicopter emergency medical service's response to winch missions in Victoria, Australia. *Prehospital Emerg. Care* **2016**, *20*, 106–110.
9. Heschl, S.; Andrew, E.; de Wit, A.; Bernard, S.; Kennedy, M.; Smith, K.; Study Investigators. Prehospital transfusion of red cell concentrates in a paramedic-staffed helicopter emergency medical service. *Emerg. Med. Australas.* **2018**, *30*, 236–241.
10. Mikkelsen, S.; Krüger, A.J.; Zwisler, S.T.; Brøchner, A.C. Outcome following physician supervised prehospital resuscitation: A retrospective study. *BMJ Open* **2015**, *5*, e006167
11. Tissier, C.; Bonithon-Kopp, C.; Freysz, M.; French Intensive care Recorded in Severe Trauma (FIRST) study group. Statement of severe trauma management in France: Teachings of the FIRST study. In *Annales Françaises D'anesthésie et de Réanimation*; Elsevier Masson: Amsterdam, The Netherlands, 2013; Volume 32, pp. 465–471.
12. Nielsen, K.; Hansen, C.M.; Rasmussen, L.S. Airway management in unconscious non-trauma patients. *Emerg. Med. J.* **2012**, *29*, 887–889.
13. Otsuka, H.; Sato, T.; Morita, S.; Nakagawa, Y.; Inokuchi, S. A case of blunt traumatic cardiac tamponade successfully treated by out-of-hospital pericardial drainage in a "doctor-helicopter" ambulance staffed by skilled emergency physicians. *Tokai J. Exp. Clin. Med.* **2016**, *41*, 1–3.
